# Activation of neutral sphingomyelinase 2 through hyperglycemia contributes to endothelial apoptosis via vesicle-bound intercellular transfer of ceramides

**DOI:** 10.1007/s00018-021-04049-5

**Published:** 2021-12-24

**Authors:** Andreas Zietzer, Alina Lisann Jahnel, Marko Bulic, Katharina Gutbrod, Philip Düsing, Mohammed Rabiul Hosen, Peter Dörmann, Nikos Werner, Georg Nickenig, Felix Jansen

**Affiliations:** 1 Department of Internal Medicine II, University Hospital Bonn, University of Bonn, Venusberg-Campus 1, 53127 Bonn, Germany; 2 Institute of Molecular Physiology and Biotechnology of Plants, University of Bonn, Karlrobert-Kreiten-Str. 13, 53115 Bonn, Germany; 3 Krankenhaus der Barmherzigen Brüder Trier, Nordallee 1, 54292 Trier, Germany

**Keywords:** Extracellular vesicles, Diabetes, Endothelial cell, Apoptosis, Ceramide, SMPD3

## Abstract

**Background:**

Pro-apoptotic and pro-inflammatory ceramides are crucially involved in atherosclerotic plaque development. Local cellular ceramide accumulation mediates endothelial apoptosis, especially in type 2 diabetes mellitus, which is a major cardiovascular risk factor. In recent years, large extracellular vesicles (lEVs) have been identified as an important means of intercellular communication and as regulators of cardiovascular health and disease. A potential role for lEVs as vehicles for ceramide transfer and inductors of diabetes-associated endothelial apoptosis has never been investigated.

**Methods and Results:**

A mass-spectrometric analysis of human coronary artery endothelial cells (HCAECs) and their lEVs revealed C16 ceramide (d18:1–16:0) to be the most abundant ceramide in lEVs and to be significantly increased in lEVs after hyperglycemic injury to HCAECs. The increased packaging of ceramide into lEVs after hyperglycemic injury was shown to be dependent on neutral sphingomyelinase 2 (nSMase2), which was upregulated in glucose-treated HCAECs. lEVs from hyperglycemic HCAECs induced apoptosis in the recipient HCAECs compared to native lEVs from untreated HCAECs. Similarly, lEVs from hyperglycemic mice after streptozotocin injection induced higher rates of apoptosis in murine endothelial cells compared to lEVs from normoglycemic mice. To generate lEVs with high levels of C16 ceramide, ceramide was applied exogenously and shown to be effectively packaged into the lEVs, which then induced apoptosis in lEV-recipient HCAECs via activation of caspase 3. Intercellular transfer of ceramide through lEVs was confirmed by use of a fluorescently labeled ceramide analogue. Treatment of HCAECs with a pharmacological inhibitor of nSMases (GW4869) or siRNA-mediated downregulation of nSMase2 abrogated the glucose-mediated effect on apoptosis in lEV-recipient cells. In contrast, for small EVs (sEVs), hyperglycemic injury or GW4869 treatment had no effect on apoptosis induction in sEV-recipient cells.

**Conclusion:**

lEVs mediate the induction of apoptosis in endothelial cells in response to hyperglycemic injury through intercellular transfer of ceramides.

**Graphical abstract:**

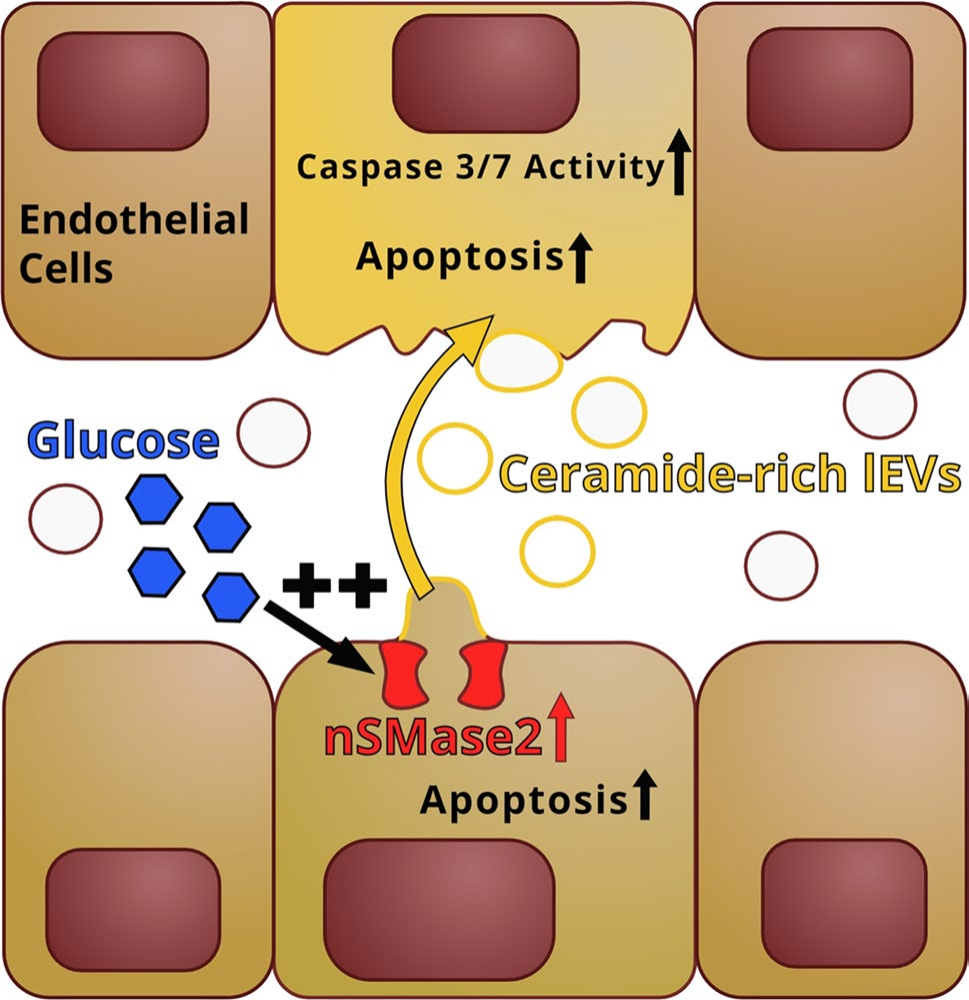

**Supplementary Information:**

The online version contains supplementary material available at 10.1007/s00018-021-04049-5.

## Background

In recent years, sphingolipids have been shown to be important mediators of cardiovascular health and disease [1]. Elevated levels of ceramides in human plasma have been linked to an increased risk of cardiovascular events [2]. In particular, high levels of ceramides with a long carboxyl chain (e.g., C16 ceramide) have been shown to be associated with the development of cardiovascular disease and to be of prognostic value for the prediction of cardiovascular events [3]. On the cellular level, ceramides are produced in response to toxic stimuli and serve as potent pro-apoptotic and pro-inflammatory signaling molecules [4–6]. Generally, cellular ceramide production is governed by sphingomyelinases and ceramidases [7, 8]. Rapid generation of ceramide, in response to cellular injury from extracellular stressors, is mainly regulated by sphingomyelinases. Of these sphingomyelinases, isoform 3 (sphingomyelin phosphodiesterase 3 = *SMPD3*, which codes for the protein neutral sphingomyelinase 2 = nSMase2) is the most responsive isoform with regard to important inductors of atherosclerotic plaque development, such as oxidative stress, inflammation, and apoptosis [9, 10]. Therefore, nSMase2 activity has been shown to be involved in atherosclerotic plaque development in vivo [11]. Moreover, ceramides have been shown to be involved in the development of type 2 diabetes mellitus [12], which is one of the most important risk factors for cardiovascular disease [13, 14]. Diabetes-associated hyperglycemia also triggers atherosclerotic plaque development through the impairment of endothelial function and regeneration [15, 16]. This again involves the induction of endothelial apoptosis through oxidative stress and the accumulation of ceramides [17–20].

Over the past decade, extracellular vesicles (EVs) have been identified as crucial regulators of atherosclerotic plaque development [21–24]. Virtually every kind of cell releases EVs, which can be taken up by both adjacent and distant cells [25, 26]. Therefore, EVs have the potential to transport various molecules, such as nucleic acids and proteins, but also lipids [21]. The relatively small encapsulated volume, in relation to the large surface of the outer membrane, makes EVs interesting as lipid carriers, and the strong curvature of the EV membrane requires lipids with a specific head-group-to-tail size ratio [27]. Ceramides typically induce membrane curvature due to their conical shape, which is why the release of small EVs (exosomes) from the multivesicular endosome is dependent on the supply of ceramide provided by nSMases [28, 29]. For large EVs (lEVs, formerly called microvesicles), which are released via direct budding from the cytoplasmic membrane, the importance of the ceramide supply and sphingomyelinase activity remains controversial [30, 31]. In the context of cardiovascular health, however, lEVs are by no means less important than small EVs, as ours and other groups have shown [32–34].

In a recent study, ceramide levels from plasma-derived EVs have been identified as biomarkers of acute myocardial infarction [35]. Mechanisms of loading ceramides into lEVs and the biological function of lEVs as intercellular ceramide transporters has not yet been investigated in the context of cardiovascular disease. Likewise, the roles of cardiovascular risk factors such as type 2 diabetes mellitus on vesicular ceramide release and on the induction of ceramide-mediated apoptosis remain unclear. In this study, we show for the first time that lEV-bound intercellular ceramide transfer is a mediator of endothelial apoptosis under hyperglycemic conditions.

## Methods

### Cell culture

As our experimental model, we used male human coronary artery endothelial cells (HCAECs) from Promocell (Cat# C-12221), which were cultured under standard conditions (37 °C, 5% CO_2,_ 100% relative humidity) using Endothelial Cell Growth Medium MV (Promocell Cat# C-22020). Experiments were performed with cells from passages 8–9. For lEV isolation, the HCAECs were cultured in serum-free medium for 24 h, similar to previously published methods from ours and other groups [36–38]. Hyperglycemic injury was induced by the addition of a 20% glucose solution (glucose 20%, B. Braun, Cat# B05BA03) to achieve a supplement of 5 mM and 30 mM glucose to the regular medium, as previously reported by our and other groups [37, 39]. This model is clinically relevant, as blood glucose concentrations higher than 30 mM can be observed in patients suffering from hyperglycemic crises, which is not a rare but severe complication of diabetes mellitus [40]. Additionally, hyperosmolar injury was simulated by the addition of a 15% mannitol solution (M15, Burg Pharma GmbH Cat# 1021) to the regular medium, to achieve a supplement of 30 mM mannitol. To generate lEVs with increased ceramide levels, we used exogenous C16 ceramide (d18:1/16:0, N-palmitoyl-D-erythro-sphingosine, Avanti Polar Lipids Cat# 860516P, short C16), because we found it to be the most abundant ceramide in HCAECs and HCAEC-derived lEVs. C16 ceramide was dissolved in dimethyl sulfoxide (DMSO, AppliChem GmbH, Cat# A3672,0100) at a concentration of 5 mM. The final concentrations used for the cell-culture experiments are indicated in the respective figures. GW4869, an inhibitor of nSMases, was dissolved in DMSO at a concentration of 5 mM and used at concentrations of 5–10 µM, as previously reported [30, 41]. For all experiments with C16 or GW4869, equal amounts of DMSO were added to the control group. For experiments with murine endothelial cells, we used male C57BL/6 mouse primary carotid artery endothelial cells (MCAEC, Cell Biologics Cat# C57-6008), which were cultured in complete mouse endothelial cell medium (Cell Biologics M1168).

### lEV isolation

**Fig. 1:**
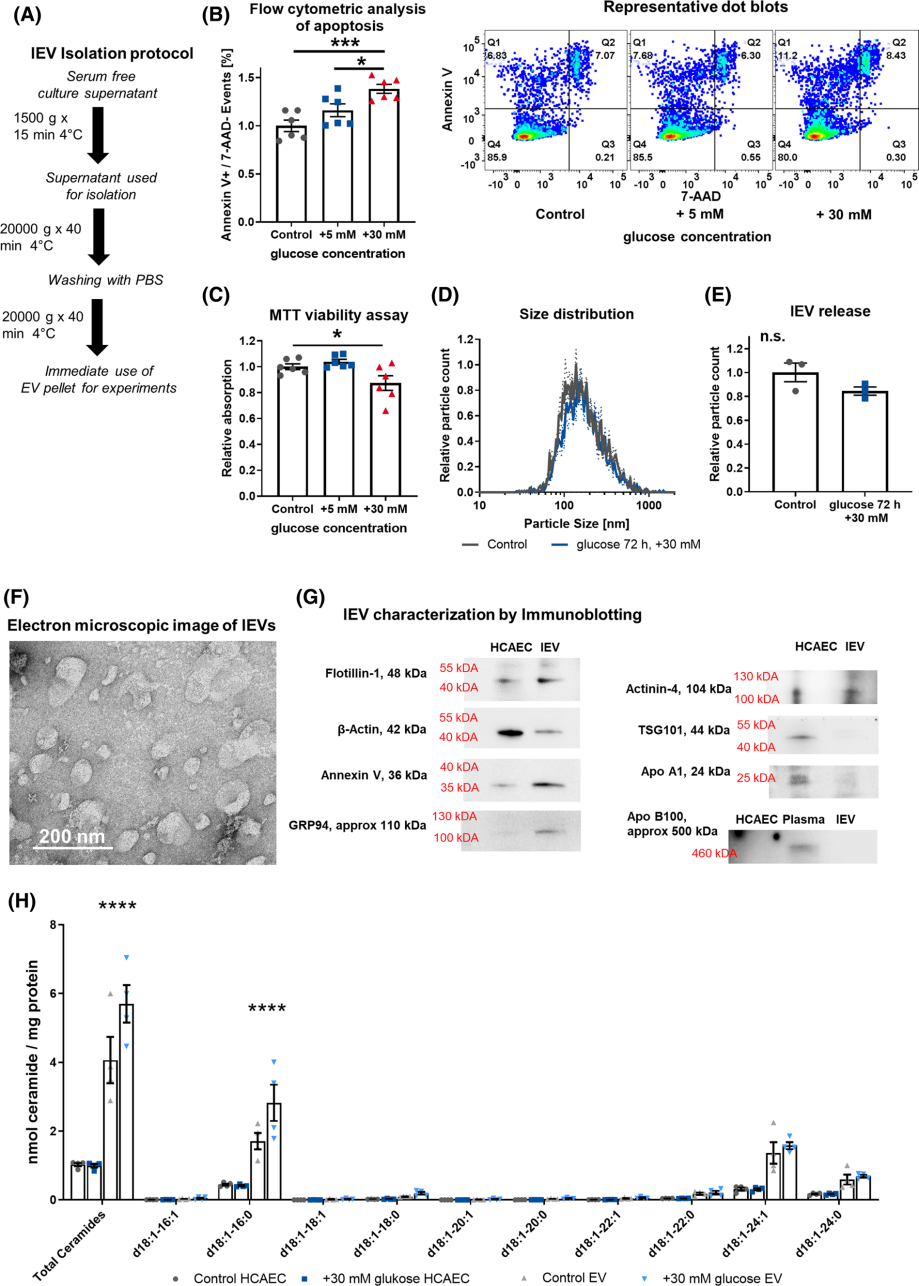
**A** Schematic diagram of the lEV isolation protocol. **B** Flow-cytometric analysis of apoptosis induction in HCAECs after hyperglycemic injury, *n* = 6, with representative dot blots. **C** MTT viability assay in HCAECs after hyperglycemic injury, *n* = 6. **D + E** Distribution of lEV size and relative lEVs released after hyperglycemic injury in HCAECs, as analyzed by nanoparticle tracking analysis, *n* = 3. **F** Direct electron microscopic imaging of HCAEC-derived lEVs with negative staining, representative image. **G** Characterization of lEVs by immunoblotting for Flotillin-1, *β*-Actin, Annexin V, GRP94, Actinin-4, TSG101, Apolipoprotein A1, Apolipoprotein B100 (with additional human plasma as an antibody control). **H** Mass spectrometric analysis of ceramides in HCAECs and lEVs after hyperglycemic injury. All data are presented as individual experiments with the mean ± SEM; *n.s.* not significant, *p < 0.05, ***p < 0.001, ****p < 0.0001. ANOVA + Bonferoni’s multiple comparison test were used for **B** + **C**, unpaired *t*-Test for **E**, two-way ANOVA + Bonferoni’s multiple comparison test  were used for **H**

The isolation of lEVs was performed as previously reported by our group [32, 37, 42], using a three-step centrifugation protocol in an Eppendorf Centrifuge 5430 with an FA-45-16-17 rotor. In brief, the culture supernatant was centrifuged at 1500×*g* for 15 min to remove cellular debris. The lEV-containing supernatant was then centrifuged at 20,000×*g* for 40 min at 4 °C to pellet the lEVs. The lEVs were washed by resuspending them in PBS and pelleted again at 20,000×*g* for 40 min (Fig. 1A). For EV stimulation experiments, equal numbers of EV-releasing cells were used across the different conditions in the same experiment. We used lEVs from approximately 2.0 × 10^6^ HCAECs after 24 h of serum-free culture and resuspended them in 2 ml of culture medium. For untreated HCAECs, this procedure led to a concentration of approximately 5.0 × 10^8^ endothelial cell-derived EVs per ml. This corresponds to the 5–15% of endothelial-cell-derived lEVs reported in the literature [43], with an estimation of 2.0 × 10^9^ – 1.0 × 10^10^ total lEVs (> 100 nm) in the peripheral blood [44–46].

### sEV isolation

sEVs were isolated from the supernatant after lEVs had been removed, as described above, through a main centrifugation step at 100,000×*g*, 4 °C for 90 min in a Beckman Coulter Optima LE-80 K ultracentrifuge with a SW41Ti rotor, as previously reported [47, 48]. Extensive characterization studies of endothelial cell-derived sEVs, isolated by this method, have been published previously by our group [48]. For stimulation experiments with sEVs, the same approach was performed as for lEVs but using sEVs from approximately 2.0 × 10^6^ HCAECs in 2 ml of culture medium, to allow for a comparison between lEVs and sEVs.

### Flow-cytometric analysis of apoptosis

Early apoptosis in HCAECs was quantified by use of the FITC Annexin V Apoptosis Detection Kit with 7-AAD (Biolegend, Cat# 640922). For this assay, we used 12-well plates at 90% confluence. The assay was carried out as recommended by the manufacturer. In brief, the cells were washed twice with PBS. Then the cells were carefully detached by use of the Detach kit (Promocell C-41210), centrifuged, and suspended in 100 µl of Annexin V binding buffer. Subsequently, the FITC-Annexin V and the 7-AAD staining solutions were added (5 µl of each), and the sample was incubated for 15 min at RT. Before analysis in a FACSCanto II (BD Bioscience) machine, another 600 µl of Annexin V binding buffer were added. For compensation, gating, and quantification we used the software FlowJo V10 (BD Bioscience), as previously reported [42]. A representative gating strategy is displayed in Figure S1.

### Nanoparticle tracking analysis

For nanoparticle tracking analysis, we used a ZetaView BASIC NTA—Nanoparticle Tracking Video Microscope PMX-120 (Particle Metrix). The analysis were performed as previously described [32]. For the analysis of lEVs from murine plasma, the lEVs from 3 µl plasma were diluted in 1000 µl PBS and analyzed by NTA. A concentration row confirmed, that the resulting concentrations were in the linear range of the NTA.

### Viability assay (MTT)

HCAEC viability was assessed by use of the MTT Cell Growth Assay Kit (Sigma-Aldrich, Cat# CT02). The assay was carried out in 96-well plates, according to the manufacturer’s recommendations. In brief, 10 µl of the AB Solution (MTT) were added to each well containing 100 µl of medium with the respective stimulant. The cells were incubated at 37 °C for 4 h. Subsequently, 100 µl isopropanol with 0.04 N HCl were added and the formazan was dissolved. Finally, absorbance was measured in a Tecan Infinite M200 Plate Reader (Tecan), using a test wavelength of 570 nm and a reference wavelength of 630 nm.

### Lipid extraction

Lipid isolation was performed as previously published [49]. In brief, approximately 1.0 × 10^7^ HCAECs and their respective lEVs were harvested with 1 ml (HCAECs) or 0.5 ml (lEVs) of a hypo-osmolar buffer containing 10 mM HEPES and 0.5 mM EDTA, together with Roche Protease Inhibitor Cocktail (Roche, Cat# 4693132001), and sonicated for 15 min in ice-cold water. The protein concentration was assessed with a Qubit-4 Fluorometer (Thermo Fisher Scientific) using the Qubit™ Protein Assay Kit (Thermo Fisher Scientific, Cat# Q33211), according to the manufacturer’s instructions. Lipid extraction was performed with a homogenous phase of chloroform and methanol (1:2, *v*/*v*), overnight at 37 °C. Subsequently, the sample was centrifuged at 2500 rpm for 15 min. The supernatant was transferred to a new container and dried under *N*_2_ flow. The extraction was then repeated with chloroform/methanol (1:1, *v*/*v*) and with chloroform/methanol (2:1, *v*/*v*), with a 1-hour incubation at 37 °C for each step. For the first analysis (glucose treatment) the procedure was followed by alkaline hydrolysis as previously described [49]. For the other experiments (addition of C16 ceramide or GW4869), the lipids were extracted by use of silica columns (Strata-1 Silica, 55 μm, 70 Å, 100 mg; Phenomenex, Aschaffenburg, Germany). To this end, the lipids were dissolved in chloroform and loaded onto the columns. The columns were washed three times with chloroform and ceramides were eluted with acetone/isopropanol (1:1, *v*/*v*) [50].

### Quantification of sphingolipids using mass spectrometry

Sphingolipid analysis was carried out using a QTRAP 6500 + LC–MS/MS system (Sciex, Darmstadt), as previously described [51].

### Immunoblotting

**Fig. 2:**
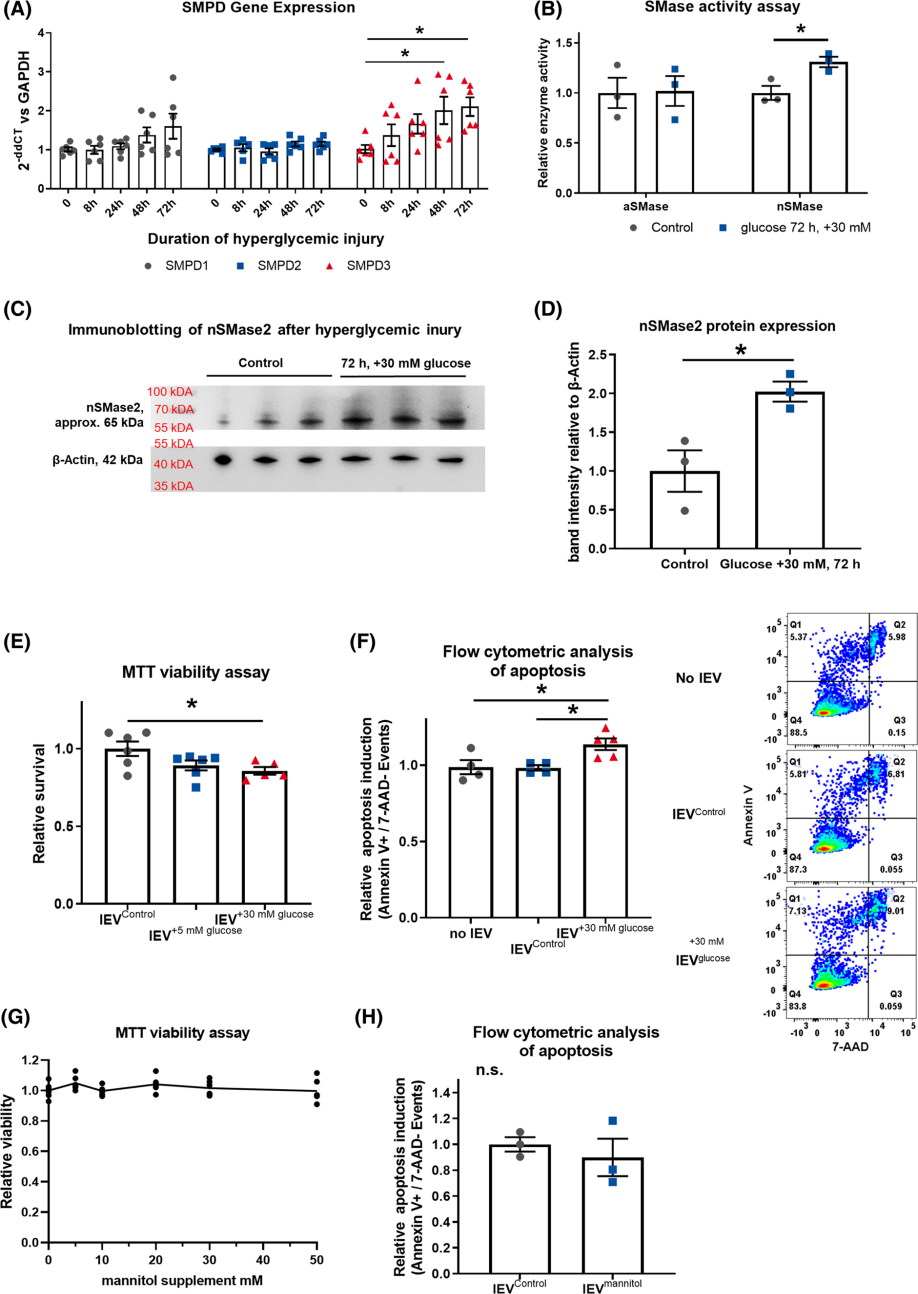
**A** Timeline of gene expression of *SMPD1-3* in HCAECs after hyperglycemic injury, presented as 2^−ddCT^ vs *GAPDH*, *n* = 6. **B** Acidic and neutral SMase enzyme activity in HCAECs after 72 h of hyperglycemic injury, *n* = 5–6. **C + D** Immunoblotting for nSMase2 and β-Actin after 72 h of hyperglycemic injury, with quantification, *n* = 3. **E** MTT viability assay in HCAECs that were treated with lEVs from HCAECs after hyperglycemic injury, *n* = 6. **F** Flow-cytometric analysis of the induction of apoptosis in HCAECs that were treated with lEVs from HCAECs after hyperglycemic injury, (right) representative dot blots, *n* = 5. **G** MTT viability assay in HCAECs after treatment with mannitol, *n* = 6. **H** Flow-cytometric analysis of the induction of apoptosis in HCAECs that were treated with lEVs from HCAECs after osmotic injury with mannitol, *n* = 3. All data are presented as individual experiments with the mean ± SEM; *n.s*. not significant, *p < 0.05, ANOVA + Bonferoni’s multiple comparison test were used for **A**, **E**, **G**, and unpaired *t*-Test for **B**, **D**, **F**, **H**

For immunoblotting of lEVs and HCAECs, the samples were lysed in RIPA buffer (Sigma-Aldrich, Cat# R0278) with 1:25 Protease Inhibitor Cocktail (Roche, Cat# 4693132001). After ultra-sonication for 10 min, the protein concentration was assessed in a Qubit-4 Fluorometer (Thermo Fisher Scientific) with the Qubit™ Protein Assay Kit (Thermo Fisher Scientific, Cat# Q33211). The assay was carried out according to the manufacturer’s instructions. 50 µg total protein (10 µg were used for the analysis of cellular lysate in Fig. 2C) from human or murine samples were diluted 2:1 in 3 × Laemmli buffer and loaded onto an SDS-PAGE gel (Biorad, Cat#456-1084 and 456-1024) using the Mini PROTEAN System (Biorad). For western blotting, we used a Roti-NC nitrocellulose membrane (Carl Roth GmbH, HP40.1). Subsequently, the membranes were blocked with 5% BSA (Sigma-Aldrich) for one hour at RT. As primary antibodies for human lEVs, we used monoclonal mouse anti-Annexin V antibody (Abcam, Cat# ab54775) 1:1000, mouse anti-Flotillin-1 antibody (BD Bioscience, Cat# 610820) 1:1000, mouse anti-GRP94 antibody (Abcam, Cat# ab210960) 1:500, recombinant rabbit monoclonal anti-TSG101 antibody (Abcam Cat# ab125011) 1:500, anti-alpha Actinin-4 antibody (Abcam Cat# ab108198) 1:500, mouse anti-Apolipoprotein B Monoclonal Antibody (Thermo Fisher Scientific, Cat# MA5-14671) 1:500, Chicken anti-ApoA1 polyclonal Antibody (Invitrogen, Cat# PA5-20721) 1:500, mouse anti-β-Actin antibody (Sigma-Aldrich, Cat# A1978) 1:2000, in BSA 5% overnight at 4 °C. For sEVs, we used anti-CD81 Monoclonal Antibody (Thermo Fisher Scientific, Cat# 10630D) 1:500, and anti-Alix Mouse mAb (Cell Signaling Technology, Cat# 2171S) 1:1000. For murine lEVs, we used rabbit monoclonal anti-Flotillin-1 antibody (Abcam Cat# ab41927) 1:1000 and rabbit polyclonal anti-Histone H3 antibody (Abcam Cat# ab1791) 1:1000, under identical conditions. For the cellular analysis of nSMase2 expression, a rabbit polyclonal anti-SMPD3 antibody (Thermo Fisher Scientific, Cat# PA5-49140) 1:1000 as well as the abovementioned anti-β-Actin antibody were used. The membranes were washed three times with 0.1% TBST and incubated with an HRP-conjugated monoclonal rat anti-mouse-IgG antibody (Sigma-Aldrich, Cat# A9044) 1:3000 or with an HRP-conjugated polyclonal goat anti-rabbit-IgG (Carl Roth, Cat# 4750.1) 1:1000 or with a goat anti-chicken IgG HRP-conjugated antibody (Abcam, Cat# ab97135) 1:3000 in 5% BSA for one hour at RT. Thereafter, the membranes were washed again two times with 0.1% TBST and once with PBS. For development, the ECL primer western blotting detection reagent (Sigma-Aldrich) was used. Image acquisition was performed by use of a ChemiDoc Imaging System (Biorad, Cat# 17001401).

### SiRNA transfection

For siRNA transfection of HCAECs, *SMPD3* siRNA (Invitrogen, Cat# AM16708, Assay ID 132606) as well as silencer negative control No. 1 siRNA (Invitrogen, Cat# AM4611) were used at a final concentration of 15 nM together with Lipofectamine RNAiMAX Transfection reagent (Invitrogen, Cat# 13778150) at a final concentration of 3.75 µl/ml for 48 h.

### Electron microscopy of lEVs

For transmission electron microscopic imaging, lEVs were harvested from one 10-cm dish (60 cm^2^ growth area), as described above. After isolation, the lEVs were resuspended in 10 µl PBS with Protease Inhibitor Cocktail (Roche, Basel, Switzerland, Cat# 4,693,132,001). 5 µl of the sample were loaded on Formvar-coated copper grids (Science Services, München, Germany) and fixed twice with 2% paraformaldehyde for 5 min and subsequently for 5 min with 1% glutaraldehyde. After extensive washing with distilled water, the sample was contrasted with 1.5% uranyl acetate for 4 min. Image acquisition was performed with a Jem-2100Plus (Jeol), which was operated at 200 kV, together with a Gatan OneView 4 K camera.

### RNA isolation and quantitative real-time PCR

RNA was isolated with Trizol (Invitrogen, Cat# 15596026) and chloroform, as previously described [47]. In brief, the cells were lysed in Trizol, after washing with ice-cold PBS. RNA was isolated with chloroform, precipitated with isopropanol, washed twice with ethanol, dried, and resuspended in water. The quality and concentration of the RNA were assessed using a Nanodrop2000 (Thermo Fisher Scientific).

For reverse transcription, we used 0.5 μg RNA and the Omniscript RT Kit (Qiagen, Cat# 205111) with a “random” primer (Roche, Cat# 11034731001), following the manufacturer’s protocol. For real-time PCR, we diluted 20 µl cDNA preparation with 480 µl water and used 9 µl of the dilution together with 11 µl of the Taqman Gene Expression Master Mix (Applied Biosystems, Cat# 4440040), and the respective Taqman probes for *SMPD1*, *SMPD2*, *SMPD3*, *CERS5*, *CERS6*, and *GAPDH* (all from Thermo Fisher Scientific: SMPD1 Cat# 4331182, Assay ID Hs04183448_m1; SMPD2, Cat# 4331182, Assay ID Hs00162006_m1; SMPD3, Cat# 4331182, Assay ID Hs00920354_m1; CerS5, Cat# 4331182, Assay ID Hs00332291_m1; CerS6, Cat# 4331182, Assay ID Hs00826756_m1; GAPDH, Cat# 4331182, Assay ID Hs02786624_g1) in a 7500 HT real-time PCR instrument (Applied Biosystems). We used *GAPDH* as an internal control and relative expression was calculated as 2^−ΔΔCT^.

### Sphingomyelinase activity assays

For the assessment of nSMase activity, a Sphingomyelinase Assay Kit (Abcam Cat# ab138876) was used according to the manufacturer’s instructions. In brief, cellular lysates from HCAECs were prepared using Mammalian Cell Lysis Buffer (Abcam Cat# ab179835). The protein concentration of the lysates was assessed as described above. 5 mg of total protein in 50 µl were used for the assay together with SMase reaction buffer and sphingomyelin (both part of the kit) for 2 hours at 37 °C to allow for hydrolysis of sphingomyelin. Phosphocholine production was then quantified with the AbBlue Indicator system, by measuring the absorbance of the samples at 655 nm in a Tecan Infinite M200 Plate Reader (Tecan). Sphingomyelinase activity was calculated by use of a standard, which was provided with the kit.

For the assessment of acidic sphingomylinase activity, the Acidic Sphingomyelinase Assay Kit (Abcam, ab190554) was used according to the manufacturer’s instructions. Like for the nSMase assay, HCAECs were lysed in Mammalian Cell Lysis Buffer (Abcam, Cat# ab179835) and the protein concentration was assessed. For the aSMase assay, the protein concentration was adjusted to 80 µg/µl and 50 µl were used for incubation with the respective buffers for 2 hours at 37 °C to allow hydrolysis of the sphingomyelin. Phosphocholine production was then quantified with the AbRed Indicator system by measuring the fluorescence signal (Ex/Em = 540/590 nm) in a Tecan Infinite M200 Plate Reader (Tecan). ASMase enzyme activity was calculated as relative activity normalized to the untreated sample.

### Caspase 3/7 assay

Caspase 3/7 activity was assessed by use of the CellEvent Caspase-3/7 Green Detection Reagent (Thermo Fisher Scientific, Cat # C10423) in a 96-well plate after stimulation with 5.0 × 10^8^ /ml lEVs for 24 h. The assay was then carried out as recommended by the manufacturer, and the plates were imaged with a Zeiss Axio Observer microscope and analyzed with the ZEN 2.3 pro software.

### Apoptosis proteome profiler array

The Proteome Profiler Human Apoptosis Array Kit (R&D Systems Cat#ARY009) was used according to the manufacturer’s instructions. We used 300 µg total protein lysate from HCAECs after stimulation with 5.0 × 10^8^/ml lEVs for 24 h. Image acquisition was performed by use of a ChemiDoc Imaging System (Biorad, Cat# 17001401).

### Animals

All animal experiments were carried out in accordance with the animal protection law stated in the German civil code. All investigations were approved by the National Office for Nature, Environment and Consumer Protection in Recklinghausen, Nordrhein-Westfalen. All procedures conformed to the guidelines from Directive 2010/63/EU of the European Parliament on the protection of animals used for scientific purposes. We used *n* = 10, 10-week-old male C57BL6-J mice, which were purchased from Janvier Labs. To induce hyperglycemia, we used a standard streptozotocin (STZ) injection protocol from the Diabetic Complications Consortium [52]. The mice were randomly divided into two groups of which the first was injected with 150 mg/kg bodyweight of STZ in a 29.4 mg/ml sodium citrate buffer after 6 h of starvation or with sodium citrate buffer only (control animals). Before the injection, blood sugar levels were confirmed to be in the normal range, with an average of 8.3 mmol/L, as previously reported for C57BL6-J mice [53]. After 21 days, the blood sugar was assessed again to confirm hyperglycemia and the mice were euthanized by cervical dislocation. Blood was immediately drawn from the inferior caval vein into a sodium-citrate-containing syringe to give a final concentration of 3.2% citrate. The blood was then centrifuged in an Eppendorf Centrifuge 5430 with a Rotor FA-45-24-11-HS to remove blood cells and platelets: 15 min at 1500×*g* at RT, 15 min at 2000×*g* at RT, and subsequently for 2 min at 13,000×*g* at RT. The platelet-free plasma was then snap frozen in liquid nitrogen and stored at − 80 °C. lEVs were isolated from 250 µl of platelet-free plasma by centrifuging twice at 20,000×*g* for 40 min. The lEVs were then resuspended in 250 µl complete-mouse endothelial-cell medium to achieve the same concentrations of lEVs as in the murine blood. The lEV suspension was then incubated with MCAECs in a 24-well plate at 90% confluence. After 24 h, apoptosis of the MCAECs was assessed using a FITC Annexin V Apoptosis Detection Kit with 7-AAD, as described above, the only adaptation being the use of 2 µl Annexin V staining solution and 1 µl 7-AAD staining solution.

### NBD-C12 transfer experiment

To assess the transfer of ceramides by lEVs among HCAECs, we used NBD-C12-ceramide (NBD-C12, Avanti Polar Lipids, Cat# 810211P-1mg). NBD-C12 ceramide was dissolved in DMSO at a concentration of 5 mM and incubated with HCAECs at concentrations of 10 or 20 µM, or the respective amounts of DMSO were added as a control, for 24 h to allow for uptake. To control the effective uptake, the cells were washed three times with warm PBS to remove excess NBD-C12, fixed with 4% PFA for 30 min at 4 °C and counterstained with VECTASHIELD mounting medium with DAPI (Vector Laboratories, Cat# H-1200). For the transfer experiment, HCAECs were treated with NBD-C12 ceramide at 10 or 20 µM for 24 h. After extensive washing to remove excess NBD-C12, the cells were incubated for 24 h with serum-free medium to trigger lEV release. From the medium, lEVs were isolated as described above. Additionally, the spontaneous formation of NBD-C12 ceramide micelles, which could be co-isolated with lEVs, was investigated. To this end, 2 ml of a 20 µM NBD-C12 solution in PBS were subjected to the same centrifugation steps as for lEV isolation. The isolated lEVs or “pseudoEVs” from NBD-C12 PBS were incubated with native HCAECs for 12 h to allow for uptake. Subsequently, the cells were washed three times extensively with PBS, fixed with 4% PFA for 30 min at 4 °C, and counterstained with VECTASHIELD mounting medium with DAPI (Vector Laboratories, Cat# H-1200). Images were acquired by use of a Zeiss Axio Observer microscope and analyzed with the ZEN 2.3 pro software.

### Quantification and statistical analysis

Data are presented as the mean ± standard error of the mean (SEM) throughout the manuscript. The number of independent replications of the respective experiment is reported as a number, *n*, in the figure legends. All statistical analyses were performed with the software Prism8. Statistical details are displayed in the figure legends. Means of two groups were compared with an unpaired *t*-test. Means of more than two groups were compared by a one-way ANOVA and Bonferoni’s post hoc test. To account for multiple testing, a two-way ANOVA and Bonferoni’s post hoc test were applied for mass spectrometric analyses of sphingolipids. All reported p values are two-sided.

## Results

### Hyperglycemic injury induces endothelial-cell apoptosis and enhances ceramide export into large extracellular vesicles

To simulate diabetic conditions in vitro, HCAECs were incubated with regular medium that was supplemented with 5 or 30 mM glucose. While adding 5 mM glucose did not have any effect on cell viability or the induction of apoptosis, the addition of 30 mM glucose for 72 h led to a decline of HCAEC viability and an induction of apoptosis (Fig. 1B + C). After 24 h of serum-free culture, lEVs were isolated from the HCAECs and characterized according to the recommendations of the International Society for Extracellular Vesicles (ISEV) [54]. As shown by nanoparticle tracking analysis, high concentrations of glucose did not significantly change the release or size distribution of lEVs (Fig. 1D + E). Electron microscopic imaging showed typical, cup-shaped lEVs mainly 100–200 nm in size and immunoblotting confirmed the presence of Annexin V, Flotillin-1, Actinin-4, and GRP94 as markers for EVs. (Fig. 1F + G). The cytoplasmic protein β-Actin, the ESCRT member TSG101, which is enriched in small EVs, as well as the Apolipoproteins A1 and B100 were only present in traces. To evaluate the effect of hyperglycemic injury on vesicular ceramide export, we performed a mass spectrometric analysis of ceramides from lEVs and HCAECs after hyperglycemic injury (Fig. 1H). We found that injury with an addition of 30 mM glucose for 72 h led to an increase of the total ceramide levels in lEVs. The increase in lEV ceramide levels was driven by a significant increase in d18:1/16:0 ceramide (C16), which was also the most abundant ceramide in non-glucose-treated HCAEC-derived lEVs. Interestingly, cellular ceramide levels were not changed significantly after glucose injury (Fig. 1H). Besides ceramides, we also found dihydroceramides to be significantly increased in lEVs, however at much lower levels. Sphingomyelin levels in lEVs were not significantly changed in contrast to cellular sphingomyelins, which were significantly reduced after hyperglycemic injury (Figure S2).

### Glucose injury triggers SMPD3 activation and leads to increased apoptosis in lEV recipient cells

As sphingomyelinases have been shown to be involved in EV release [30], we evaluated the effect of hyperglycemic injury on SMPD1-3 expression in a time-course experiment. We found that *SMPD3* RNA expression was significantly upregulated after 48 h and 72 h of incubation with + 30 mM glucose, while expression of *SMPD1* and *SMPD2* RNA were unchanged (Fig. 2A). The increased expression of *SMPD3* was mirrored by a significantly elevated enzyme activity of nSMase and protein expression in the cellular lysate, while acidic sphingomyelinase activity remained unchanged (Fig. 2B-D). As the vesicular export of C16 ceramide (d18:1–16:0) was most significantly changed by glucose injury, we also investigated, if the expression of ceramide synthase 5 and 6 (CerS5 + CerS6) would be altered, because these two enzymes govern the de novo synthesis of C16 ceramide. No differences were detected in *CerS5* or *CerS6* expression after 72 h of hyperglycemic injury in HCAECs (Figure S3A). This indicates that in our model the sphingomyelinase pathway is activated, whereas expression of the de novo synthesis pathway remains unchanged. The lEVs from HCAECs after hyperglycemic injury were then used to treat native HCAECs, to investigate the effect of the glucose treatment on the ability of the lEVs to change the phenotype of a recipient cell. What we found was that the ceramide rich lEVs from hyperglycemic HCAECs significantly reduced viability and increased apoptosis in lEV-treated HCAECs (Fig. 2E + F). Prior degradation of the EVs with Triton-X completely abrogated the pro-apoptotic effect of the EVs (Figure S4). To exclude that the observed effects of glucose on *SMPD3* expression and apoptosis induction in lEV recipient cells were due to unspecific osmotic stress, we repeated the key experiments with a mannitol supplement of 30 mM to have identical osmotic conditions. Mannitol treatment neither reduced HCAEC viability, nor induced apoptosis in EV-recipient cells (Fig. 2G + H). Similarly, the mRNA expression levels of sphingomyelinases or ceramide synthases remained unchanged after osmotic injury with mannitol (Figure S3B). This result indicates, that the observed effect of glucose on *SMPD3* activation and apoptosis induction through vesicular ceramide transfer is independent from osmotic injury.

### lEVs from hyperglycemic mice induce apoptosis in MCAECs

**Fig. 3:**
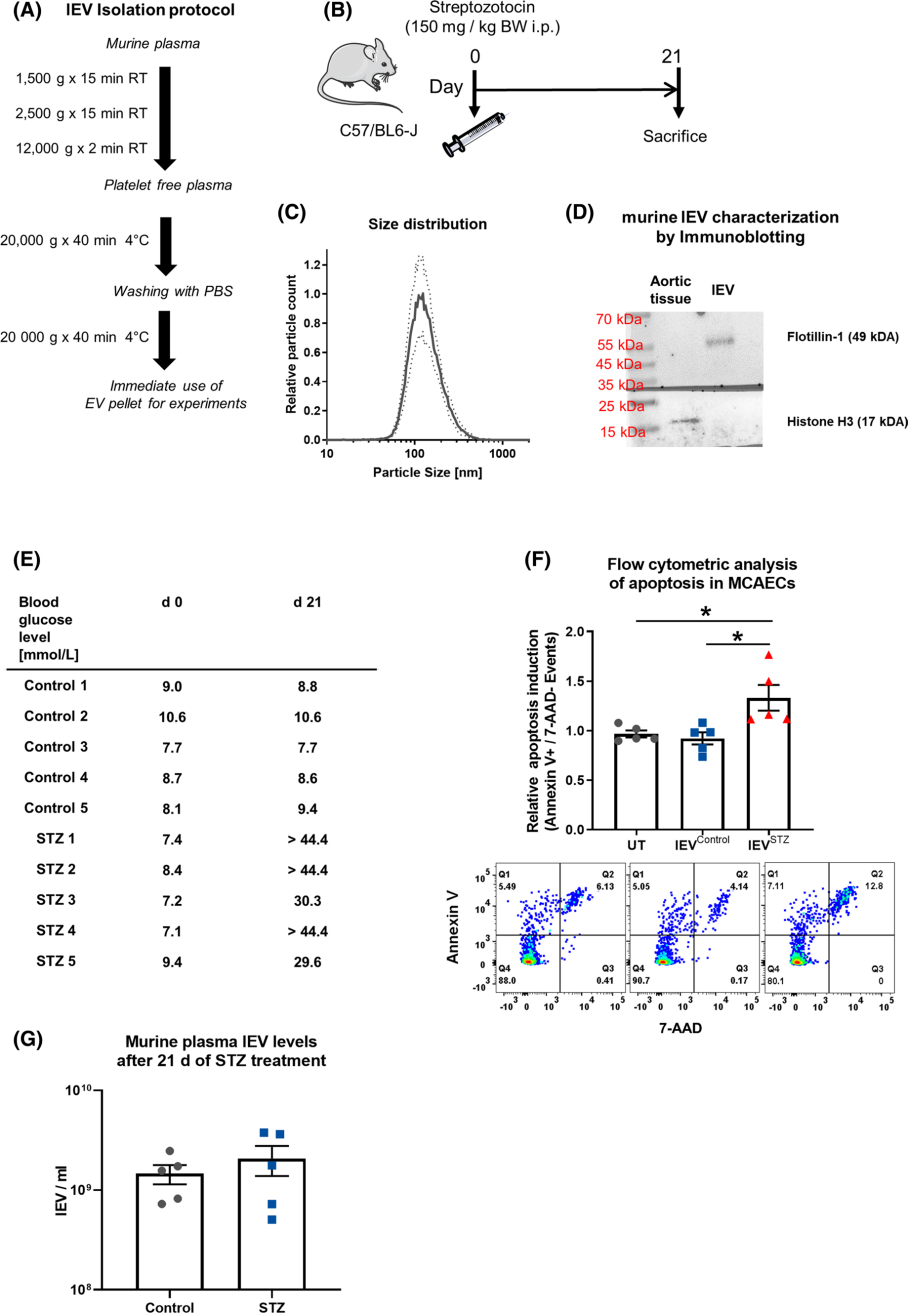
**A** Schematic diagram of the lEV isolation protocol from murine blood. **B** Diagram of the experimental set-up of the mouse experiment to induce hyperglycemia through streptozotocin injection. **C** Analysis of murine lEV size distribution by nanoparticle tracking analysis, *n* = 3. **D** Characterization of lEVs by immunoblotting for Histone H3 and Flotillin-1. **E** Table of individual blood sugar levels of the mice. **F** Flow-cytometric analysis of the induction of apoptosis in MCAECs that were treated with lEVs from hyperglycemic and normoglycemic mice, *n* = 5. **G** Nanoparticle tracking analysis of lEV concentration in murine plasma from control animals and STZ-treated animals, *n* = 5. *p < 0.05, ANOVA + Bonferoni’s multiple comparison test was used for **E**

To confirm the pro-apoptotic effect of lEVs from hyperglycemic conditions, we performed an ex vivo experiment, for which we induced hyperglycemia in mice through the injection of STZ. From those mice, lEVs were isolated by differential centrifugation and characterized by NTA and immunoblotting, which confirmed a comparable size distribution as for the HCAEC-derived EVs and the presence of Flotillin-1, whereas the nuclear protein Histone 3 was absent in the murine lEVs (Fig. 3A–D). The STZ-treated mice were confirmed to be hyperglycemic, 21 days after the injection (Fig. 3E). Incubation of MCAECs with murine lEVs from hyperglycemic mice led to significantly higher levels of apoptosis than incubation with lEVs from normoglycemic mice or no lEVs (Fig. 3F). No difference in the levels of lEVs in the blood was detected after 21 days of STZ treatment (Fig. 3G).

### Externally applied ceramide is exported in lEVs and induces apoptosis in lEV recipient cells

**Fig. 4:**
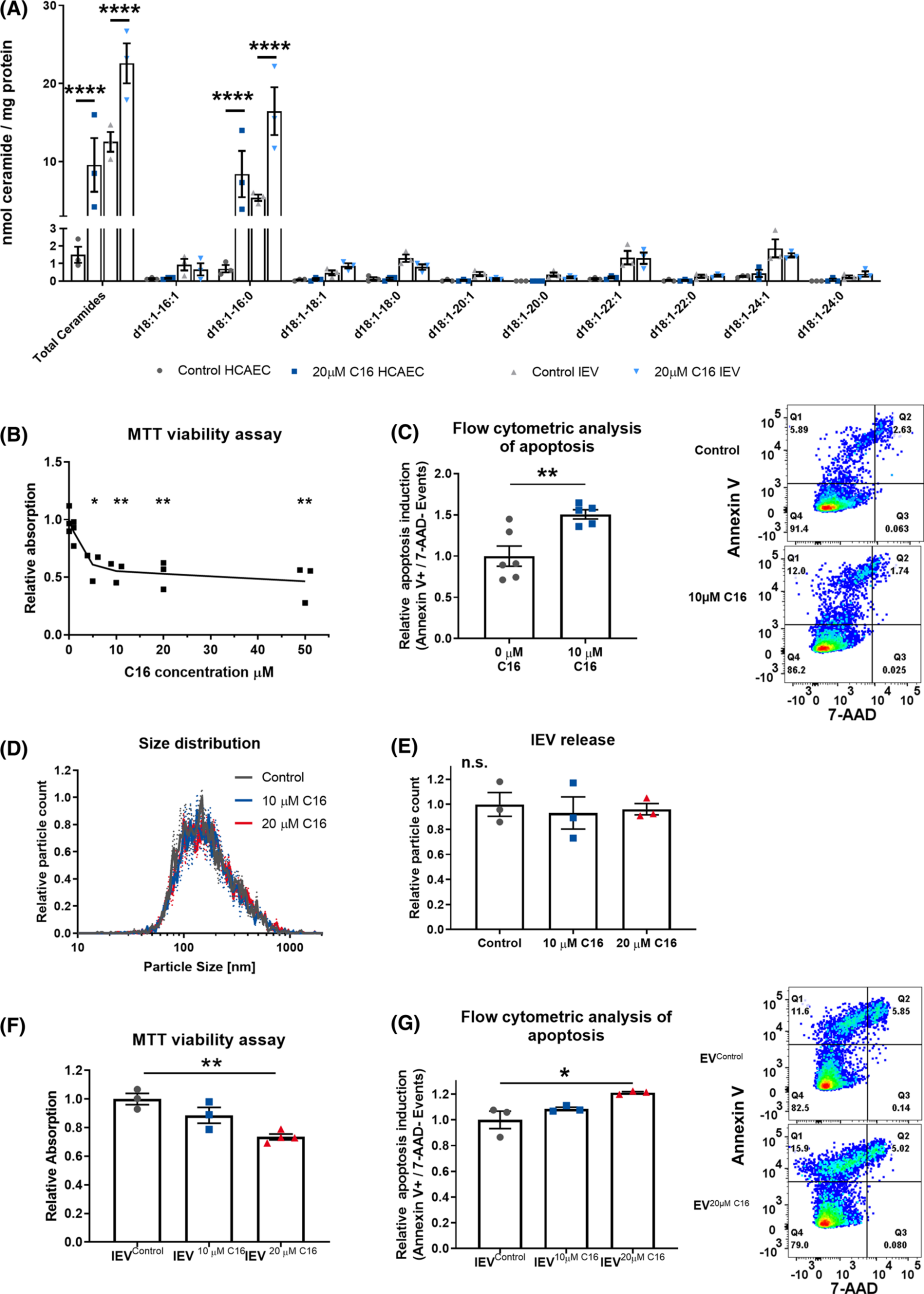
**A** Mass spectrometric analysis of ceramides in HCAECs and lEVs after treatment with 20 µM C16 ceramide for 24 h, *n* = 3. **B** MTT viability assay in HCAECs after treatment with C16 ceramide for 24 h, *n* = 6. **C** Flow-cytometric analysis of the induction of apoptosis in HCAECs after treatment with C16 ceramide for 24 h, (right) representative dot blots, *n* = 6. **D + E** Distribution of lEV sizes and relative lEV release after treatment of HCAECs with C16 ceramide for 24 h, as analyzed by nanoparticle tracking analysis, *n* = 3. **F** MTT viability assay in HCAECs after treatment with lEVs from HCAECs after C16 ceramide treatment, *n* = 3–4. **G** Flow-cytometric analysis of apoptosis induction in HCAECs after treatment with lEVs from HCAECs after C16 ceramide treatment with representative dot blots, *n* = 3. All data are presented as individual experiments with the mean ± SEM; *n.s.* not significant, *p < 0.05, **p < 0.01, ****p < 0.0001. An unpaired *t*-test was used for **C**; ANOVA + Bonferoni’s multiple comparison test were used for **B**, **E**–**G**; two-way ANOVA + Bonferoni’s multiple comparison test were used for **A**

To test the hypothesis, that increased ceramide levels in lEVs induce apoptosis in lEV- recipient HCAECs, we generated lEVs with artificially increased ceramide levels. To this end, we used purified C16 ceramide and applied it externally in cell culture at 20 µM for 24 h. The cellular uptake and packaging of the C16 ceramide into lEVs was confirmed by mass spectrometry (Fig. 4A). MTT viability assay and flow-cytometric analysis of the HCAECs themselves showed that exogenous C16 ceramide is a potent inductor of apoptosis and reduces cell viability in a dose-dependent manner (Fig. 4B + C). There was no effect of C16 treatment on lEV release or size distribution, as shown with nanoparticle tracking analysis (Fig. 4D + E). The lEVs released from cells treated with C16 ceramide reduced viability and increased apoptosis in the recipient HCAECs, also in a dose-dependent manner, with 10 µM C16 leading to 14.6% apoptosis and 20 µM C16 to 16.2% apoptosis in the recipient cells (Fig. 4F + G). Furthermore, caspase 3/7 activity was assessed in HCAECs after treatment with C16 ceramide-enriched lEVs (Figure S5A). Additionally, a proteome profiler array confirmed, the activation of caspase 3 and revealed increased levels of FADD, p27, and phospho-p53 in HCAECs after treatment with lEV^20 µM C16^ (Figure S5B).

### lEVs are effective transporters of ceramides between HCAECs

**Fig. 5:**
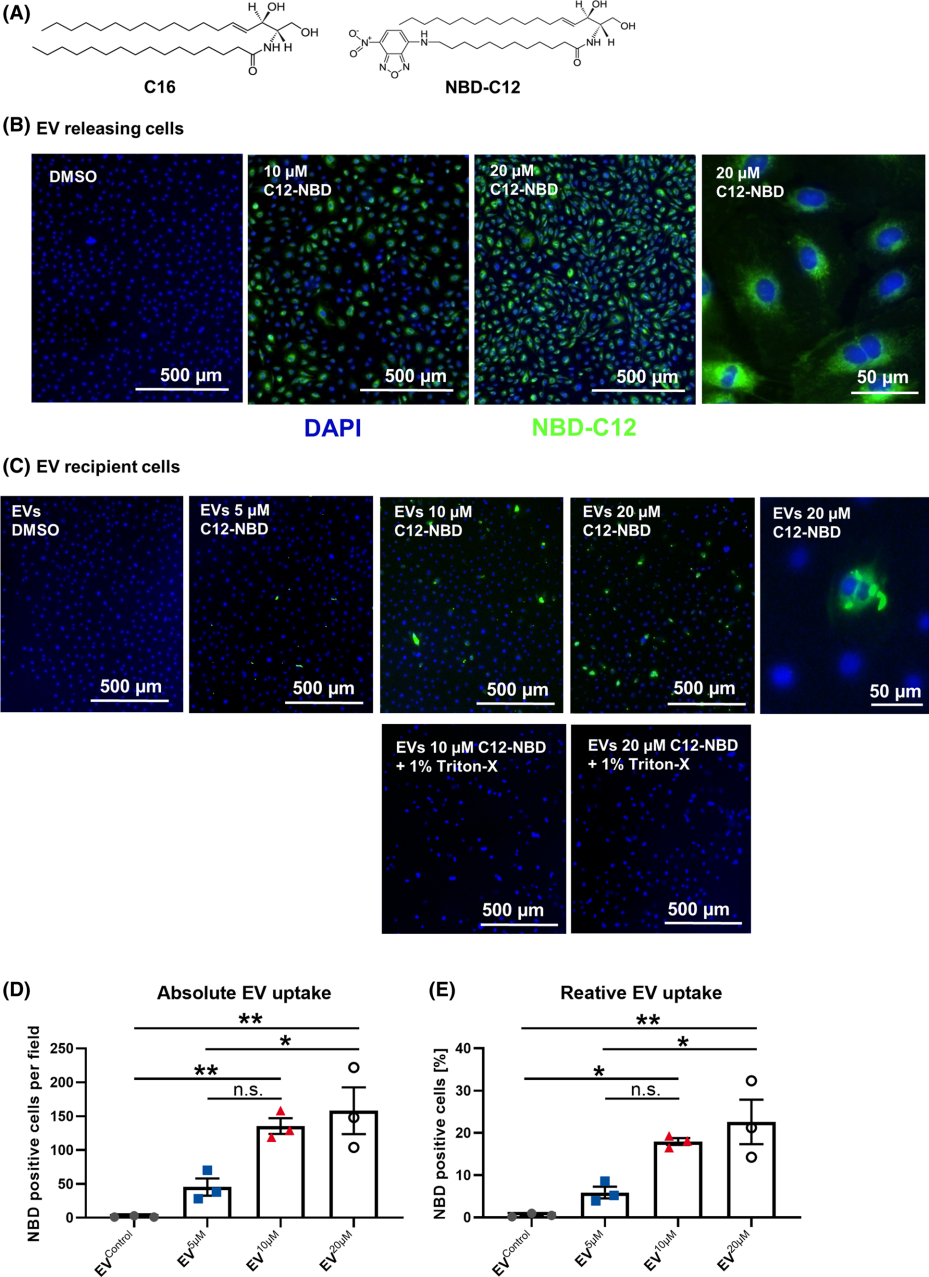
**A** Chemical structure of C16 ceramide and NBD-C12 ceramide. **B** Fluorescence microscopic imaging of NBD-C12 ceramide (green)-treated HCAECs, after counterstaining with DAPI (blue). **C** Upper row: Fluorescence microscopic imaging of HCAECs after uptake of lEVs from NBD-C12 ceramide-treated HCAECs, after counterstaining with DAPI. Lower row: Imaging of lEV-treated HCAECs, with prior lysis of the EVs in 1% Triton-X. **D + E** Absolute and relative quantification of fluorescent lEV-uptake in HCAECs, *n* = 3. All data are presented as individual experiments with the mean ± SEM; *p < 0.05, **p < 0.01, ANOVA + Bonferoni’s multiple comparison test

Next, we sought to evaluate if lEVs can effectively transfer ceramides between HCAECs. Therefore, we used a fluorescently labeled ceramide (NBD-C12 ceramide). NBD-C12 ceramide was selected because, owing to the NBD group, it has a similar acyl chain length as native C16 ceramide, which was the most abundant ceramide in HCAEC-derived lEVs (Fig. 5A). Effective uptake of NBD-C12 ceramide was achieved at doses of 10 µM and 20 µM after 24 h, as shown by fluorescence microscopy. Notably, NBD-C12 ceramide was shown to have a perinuclear distribution, indicating that intracellular localization was achieved (Fig. 5B). lEVs were isolated from NBD-C12 ceramide-treated HCAECs and incubated with native HCAECs for 12 h. In this case, the transfer of NBD-C12 ceramide was observed by fluorescence microscopic imaging. Intracellular NBD-C12 ceramide was detected in the perinuclear region of the recipient cells, similar to the distribution observed after direct exposure of the cells to NBD-C12 ceramide (Fig. 5C). We found the uptake of fluorescent lEVs by recipient cells to be dependent on the dose of NBD-C12 used to treat the lEV-releasing HCAECs, with 5 µM NBD-C12 leading to 5.9%, 10 µM leading to 17.6%, and 20 µM leading to 22.6% NBD-positive cells (Fig. 5D + E). Degradation of the lEVs by 1% Triton-X completely abolished the transfer of ceramide. Spontaneous formation of NBD-C12 micelles in an aqueous solution, that are co-isolated with lEVs, was excluded using differential centrifugation of NBD-C12 containing PBS with subsequent incubation of the isolated “pseudoEVs” with native HCAECs. No noticeable uptake of NBD-C12 “pseudoEVs” was detected (Figure S6). These experiments suggest that ceramides can be transferred between HCAECs through lEVs.

### Pharmacological inhibition of nSMase activity reduces vesicular ceramide levels and counteracts the induction of apoptosis in lEV recipient cells

**Fig. 6:**
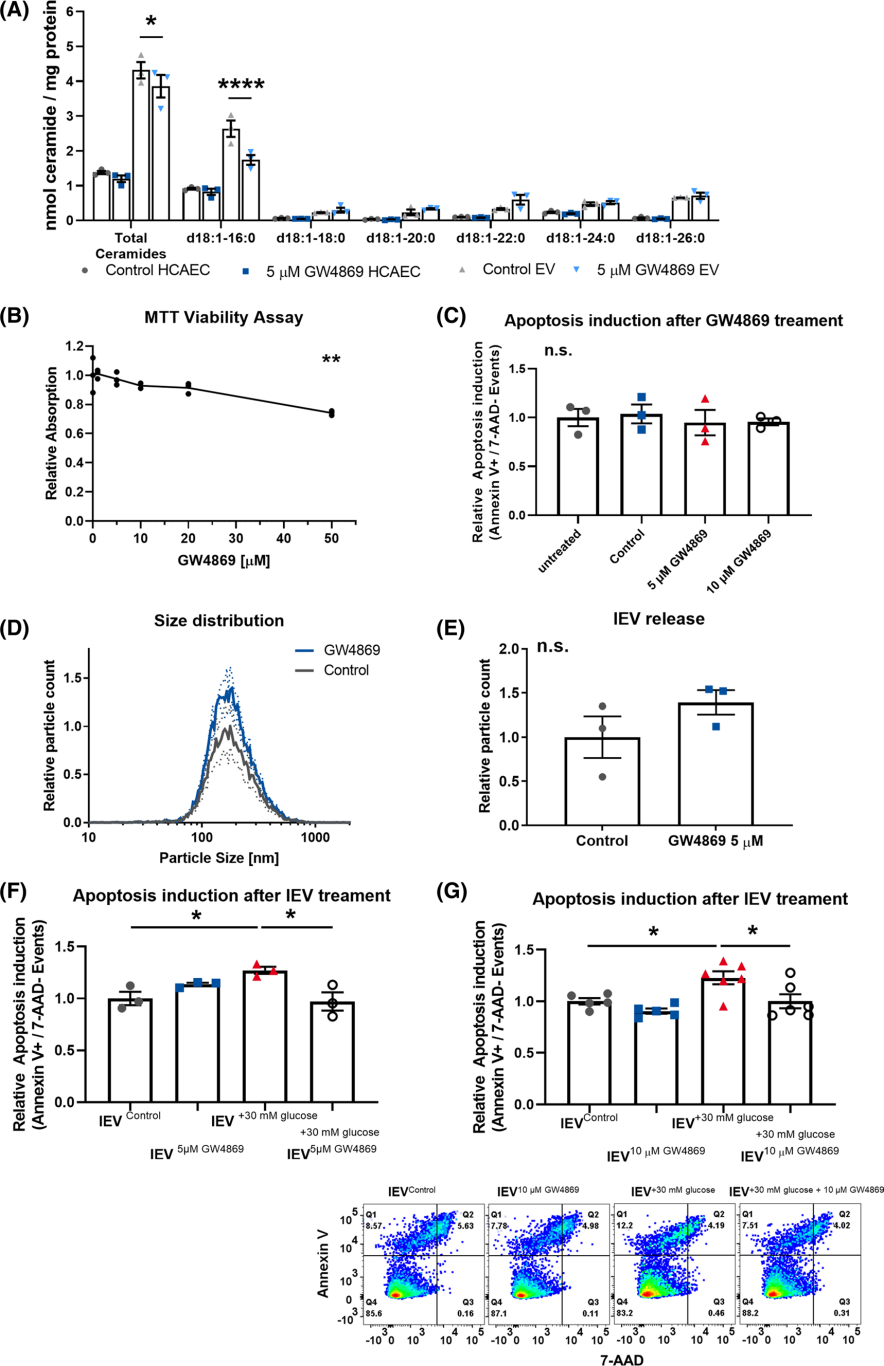
**A** Mass spectrometric analysis of ceramides in HCAECs and lEVs after treatment with 5 µM GW4869 for 24 h, *n* = 3. **B** MTT viability assay in HCAECs after treatment with GW4869 for 24 h, *n* = 2–3. **C** Flow-cytometric analysis of the induction of apoptosis in HCAECs after treatment with GW4869 for 24 h, *n* = 3. **D + E** Distribution of lEV sizes and relative lEV release after treatment of HCAECs with 5 µM GW4869 for 24 h, as analyzed by nanoparticle tracking analysis, *n* = 3. **F + G** Flow-cytometric analysis of the induction of apoptosis in HCAECs that were treated with lEVs from HCAECs after glucose injury and/or 5 µM or 10 µM GW4869 treatment, (below) representative dot blots, *n* = 3/*n* = 5–6. All data are presented as individual experiments with the mean ± SEM; *n.s.* not significant, *p < 0.05, **p < 0.01, ****p < 0.0001. An unpaired *t*-test was used for E, ANOVA + Bonferoni’s multiple comparison test were used for **B**, **C**, **F**, **G**, 2-way ANOVA + Bonferoni’s multiple comparison test were used for **A**

To confirm that packaging of ceramides into lEVs is SMase dependent, we treated HCAECs with GW4869, a pharmacological inhibitor of nSMase2. Mass spectrometric analysis of GW4869-treated lEVs revealed a significant reduction in the total ceramide levels, which was mainly driven by a reduction in C16 ceramide, again found to be the most abundant ceramide (Fig. 6A). Cell viability was not significantly reduced at concentrations of GW4869 below 20 µM and GW4869 did not cause a direct induction of apoptosis (Fig. 6B + C). Treatment of cells with 5 µM GW4869 showed a tendency towards increased lEV release, but the change was not significant (Fig. 6D + E). The combination of GW4869 with hyperglycemic injury abolished the induction of apoptosis in lEV-recipient cells at concentrations of 5 µM GW4869 and 10 µM GW4869 (Fig. 6F + G). Additionally, we investigated how glucose injury and GW4869 treatment affects the release of sEVs as well as the ability of sEVs to induce apoptosis in HCAEC-recipient cells. We found that hyperglycemia-induced SMPD3 activation increases sEV release, which could be counteracted by GW4869 treatment (Figure S7A + B). Neither hyperglycemia nor GW4869 treatment significantly changed apoptosis induction in endothelial sEV-recipient cells (Figure S7D).

### SiRNA-mediated downregulation of SMPD3 counteracts apoptosis induction after hyperglycemic injury

**Fig. 7:**
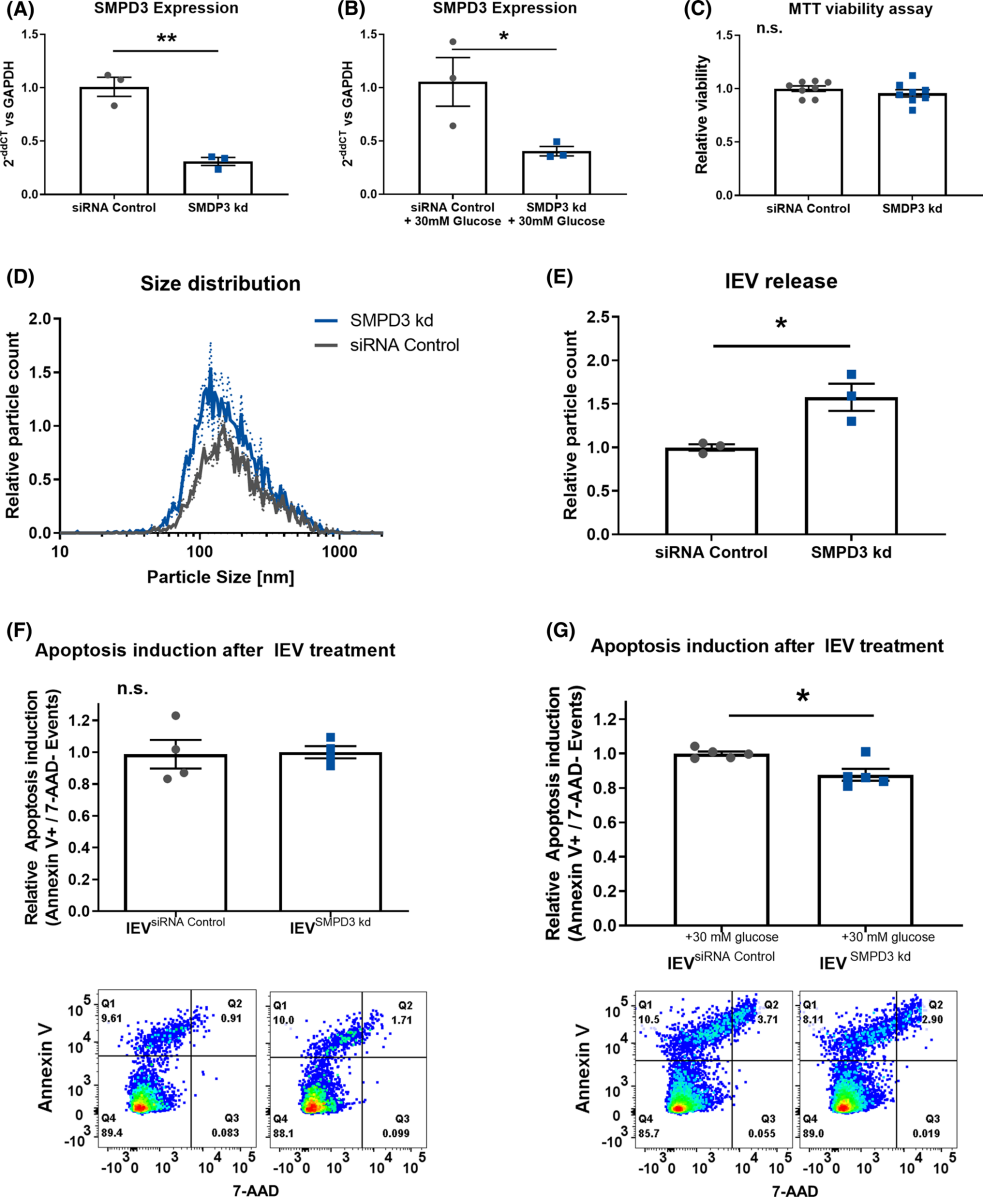
**A** + **B** Confirmation of the effective downregulation of *SMPD3* with siRNA in HCAECs under normal and hyperglycemic conditions, *n* = 3. **C** MTT viability assay in HCAECs after downregulation of *SMPD3*, *n* = 6. **D + E** Distribution of lEV sizes and relative lEV release after downregulation of *SMPD3*, as analyzed by nanoparticle tracking analysis, *n* = 3. **F + G** Flow-cytometric analysis of the induction of apoptosis in HCAECs that were treated with lEVs from HCAECs after downregulation of *SMPD3* and glucose injury, (below) representative dot blots, *n* = 4/*n* = 6. All data are presented as individual experiments with the mean ± SEM; *n.s.* not significant, *p < 0.05, **p < 0.01. An unpaired t-test was used for **A**–**G**

Additionally, we sought to determine whether the vesicular transfer of ceramides was dependent on nSMase2 activity in the EV-releasing HCAECs. Therefore, we performed siRNA-mediated downregulation of *SMPD3*. The transfection efficiency of the siRNAs was confirmed via qPCR under normal and hyperglycemic conditions (Fig. 7A + B). The viability of the HCAECs was shown to be unchanged after transfection of the siRNAs (Fig. 7C). Downregulation of *SMPD3* led to a significant increase in lEV release (Fig. 7D + E). Similar to the pharmacological inhibition by GW4869, siRNA-mediated downregulation of *SMPD3* in combination with hyperglycemic injury abolished the induction of apoptosis in HCAECs that take up lEVs (Fig. 7E + F).

## Discussion

In the present manuscript, we show for the first time that hyperglycemic injury enhances ceramide transfer in lEVs between endothelial cells. Increased packaging of ceramide in the lEVs induces apoptosis in endothelial lEV-recipient cells. Hyperglycemia activates nSMase2, which controls the pro-apoptotic function of lEVs after hyperglycemic injury.

The involvement of nSMase2 in the release of small EVs was discovered more than a decade ago [55, 56]. Consequently, the nSMase inhibitor GW4869 has been used in various studies as an inhibitor of the release of small EVs [57, 58] and has even found its way into the 2018 MISEV guidelines as an “exosome inhibitor” [54]. The effect of nSMase2 inhibition on lEV release, however, remained controversial. In an early study, inhibitors of nSMase2 did not significantly affect the release of lEVs from glial cells [59], whereas Menck et al*.* could show that inhibition of *SMPD2* and *SMPD3* gene expression as well as inhibition of nSMase activity through GW4869 enhanced lEV release from a breast cancer cell line [30]. In our study, we saw that siRNA-mediated downregulation of *SMPD3* increases lEV release from endothelial cells, which also seemed to be the case after GW4869 treatment (although borderline not significant). In contrast, enhanced nSMase2 activity after glucose injury only caused a non-significant trend towards reduced release of lEVs. nSMase2 therefore mainly controls lEV ceramide export in endothelial cells and only has a minor inhibitory effect on lEV release.

As with all lipids, the route of delivery plays an important role in the biological activity of ceramides. Ceramides that are delivered in liposomes or other complexes are several times more potent than uncomplexed, externally applied ceramides [60–62]. In our experiments, we showed that the ceramide transfer via vesicles is more effective than external application in DMSO. For the experiment in Fig. 4C, with 10 µM ceramide in 2-ml culture medium, a total of 20 nmol C16 ceramide were used. For the experiment in Fig. 4G, we can extrapolate from the mass spectrometric analysis in Fig. 4A that the EVs from 2.0 × 10^6^ HCAECs after stimulation with 20 µM ceramide as used for stimulation experiments carry 28.4 pmol C16 and 88.5 pmol total ceramide. Therefore, lEV delivery of ceramides seems more effective than applying it extracellularly. For small EVs, intercellular ceramide transfer has been assessed in the context of inflammatory liver disease and neurodegenerative disease [63–65]: For inflammatory liver disease, the authors found that hepatocytes release ceramide-rich small EVs after injury with palmitate, which triggered macrophage recruitment through chemotaxis [64]. Furthermore, ceramide-rich exosomes were shown to contribute to amyloid-induced apoptosis and to be involved in the induction of apoptosis in oligodendrocytes [63, 65]. Our work expands these findings to lEVs and to the spectrum of diabetes-induced cardiovascular disease. Of note, the induction of apoptosis after C16 ceramide treatment was more pronounced than after hyperglycemic injury, which might additionally increase the packaging of other pro-apoptotic molecules. This was not assessed in the current study. Additionally, we cannot rule out that activation of nSMase2 affects the composition of lEVs beyond the ceramide content of the lEVs, which could also explain the alteration in the induction of apoptosis in lEV-recipient cells. Considering the complex composition of lEVs, including various kinds of nucleic acids, proteins, peptides, and lipids other than sphingolipids, this question will require futher studies to be answered definitively.

In our study, the ceramide/protein ratio is higher in lEVs than in the respective EV-releasing cells (Fig. 1H). This observation confirms the results of a study from Haraszti et al*.*, who showed that microvesicles (pelleted at 10,000×*g*) from various cell types have higher ceramide/protein ratios than the respective mother cells, but also higher than exosomes, which had an even lower ceramide/protein ratio than their mother cells [27]. Thus, lEVs are potentially more effective intercellular transporters of ceramides than small EVs. Although the size distribution of our EVs does not suggest that there are large numbers of apoptotic bodies present in the samples, their presence cannot be excluded entirely, due to a lack of specific markers for apoptotic bodies.

Severe hyperglycemia (> 33 mmol/L glucose), which causes a hyperglycemic crisis, is a severe, but not rare, complication of diabetes mellitus [40]. Consequently, the addition of 30 mmol/L glucose to the culture medium is a realistic and pathophysiologically relevant model. A reduction in endothelial cell viability of 15–20% can therefore be considered a severe effect, which may contribute to the high mortality of patients with a hyperglycemic crisis [66, 67]. In accordance with our study, hyperglycemia has previously been reported to induce endothelial-cell apoptosis [19, 36, 68–70]. The implication of nSMase2 and ceramides in this process remains controversial. In contrast to our study, Ido et al*.* reported to have found no evidence for activation of ceramide producing enzymes in hyperglycemic endothelial cells [36]. This is, in our opinion, due to two key differences in the study design: First, the glucose treatment in the study by Ido and co-workers only lasted 24 h, in contrast to 72 h in our study, and second, no in-depth analysis of cellular enzyme activities was performed. A more recent study found increased aSMase activity after only 24 h of hyperglycemic injury, while again the nSMase activity was not assessed [69]. Another study reported that a reduction of the cellular ceramide levels by overexpression of acid ceramidase alleviates glucose-induced apoptosis in endothelial cells [70]. The involvement of nSMase2 in this process has not yet been reported, although ceramide accumulation is generally a well-known inductor of endothelial cell apoptosis [1, 71].

Independent of hyperglycemic injury, cellular ceramide accumulation is a strong pro-apoptotic and pro-inflammatory signal that involves the activation of p53 and the NRLP3 inflammasome [72, 73]. Furthermore, high levels of nSMase2 activity mediate TNFα-induced inflammatory processes [74]. These pathways are known to be master regulators of atherosclerotic plaque development, in which ceramides therefore contribute to the pro-inflammatory milieu [6]. Consequently, inhibition of nSMase2 is a potential target to counteract pro-inflammatory signaling in these cells [11]. In the context of cardiovascular disease, the plasma levels of long-chain ceramides have been linked to adverse events after coronary angiography [1] and cardiovascular death from coronary artery disease [2]. Vesicular ceramide levels have, however, been underappreciated in recent research. Therefore, the potential for vesicular ceramide levels to serve as biomarkers of acute myocardial infarction has only been very recently discovered [35]. Our work may contribute to this direction and highlights the importance of vesicular ceramide transfer as a mediator of diabetes-associated cardiovascular disease development.

In summary, we found evidence of a new cellular mechanism for the induction of endothelial-cell apoptosis under hyperglycemic conditions. We identified the inhibition of nSMase2 activity as a promising approach to therapeutically influence this mechanism. Additionally, we established a role for lEVs as efficient intercellular transport vehicles for ceramides.

### Supplementary Information

Below is the link to the electronic supplementary material.

Supplementary file1 (DOCX 2010 KB)

## Data Availability

All data are available from the corresponding author upon reasonable request.
