# Supplementary material for: Activation of neutral sphingomyelinase 2 through hyperglycemia contributes to endothelial apoptosis via vesicle-bound intercellular transfer of ceramides
Source: Cell Mol Life Sci. 2021 Dec 24;79(1):48. doi: 10.1007/s00018-021-04049-5 (PMC8739297; doi:10.1007/s00018-021-04049-5)
Supplement: Supplementary file 1 — Supplementary file1 (DOCX 2010 KB) [file 18_2021_4049_MOESM1_ESM.docx]

**Supplemental material**

**
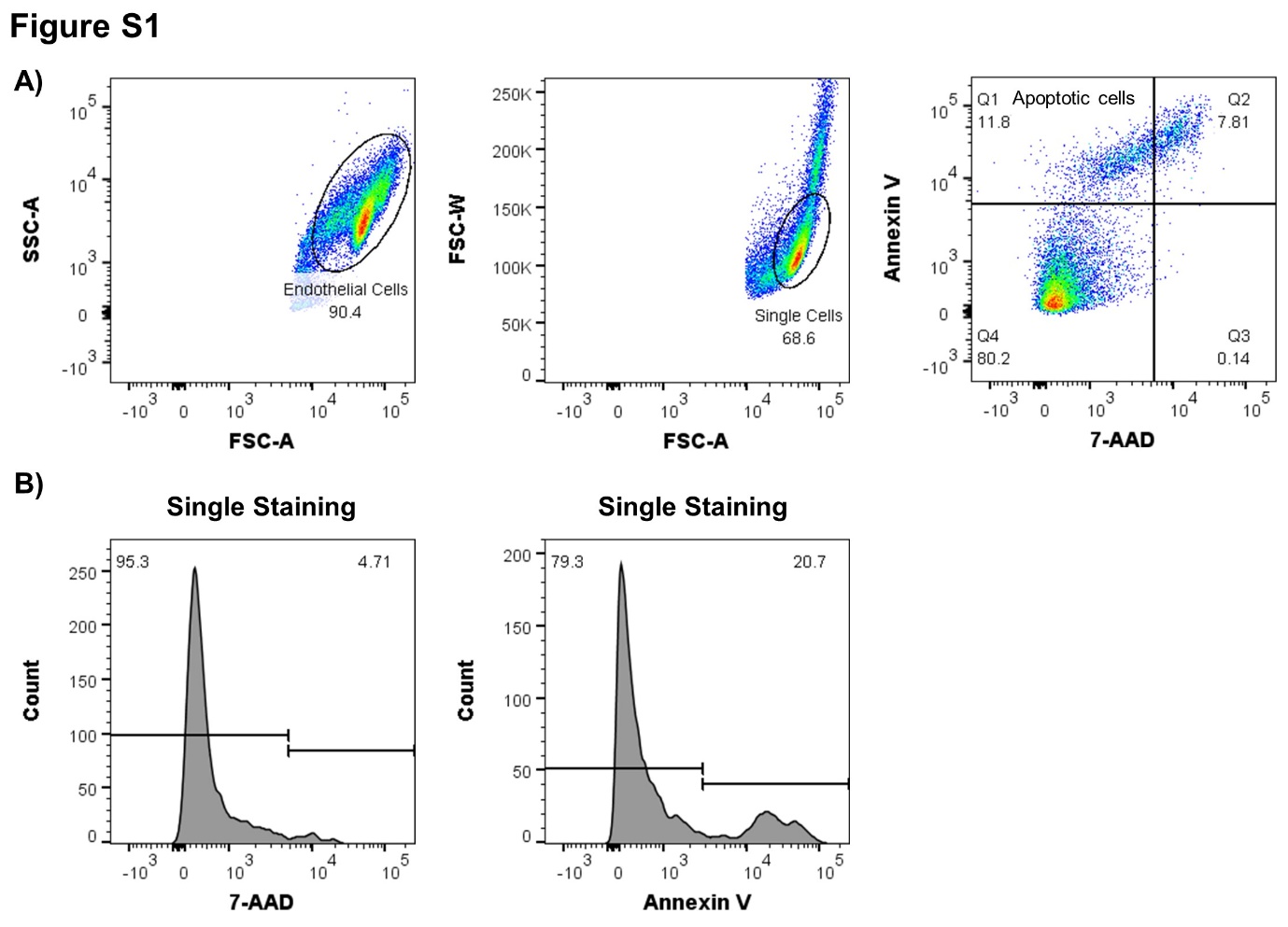
**

### **Figure S1:**

**A+B)** Representative gating strategy for the flow cytometric analysis of apoptosis, with respective single-staining measurements for 7AAD + Annexin V.

**
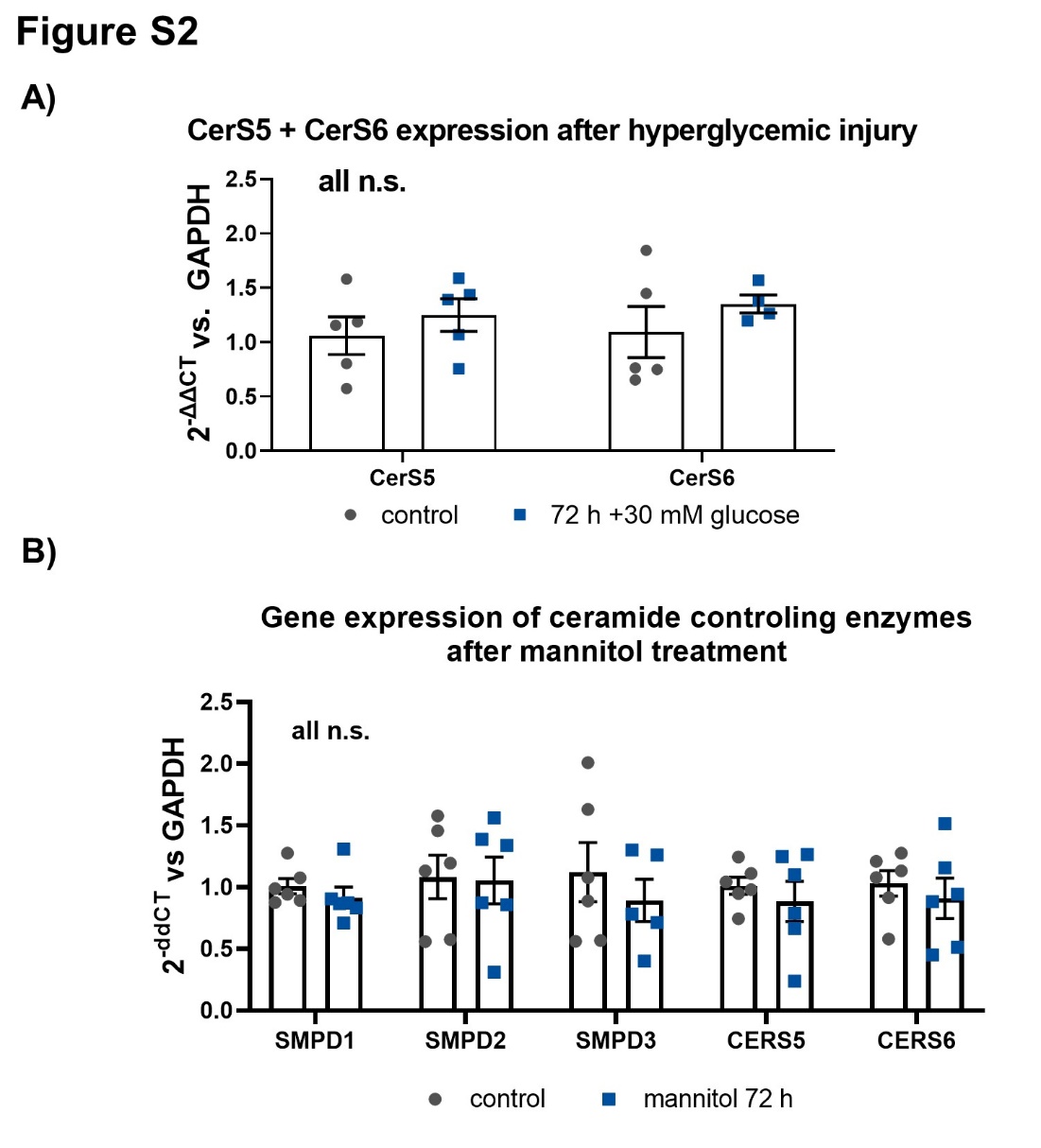
**

**Figure S2:**

**A)** Gene expression of *CerS5* and *CerS6* in HCAECs after hyperglycemic injury, presented as 2^-ddCT^ vs *GAPDH*, n=4–5. **B)** Gene expression of *SMPD1*, *SMPD2*, *SMPD3*, *CerS5*, and *CerS6* in HCAECs after osmotic injury with mannitol, presented as 2^-ddCT^ vs. *GAPDH*, n=5–6. All data are presented as individual experiments with the mean ± SEM; n.s. not significant. An unpaired t-test was used for A+B.

**
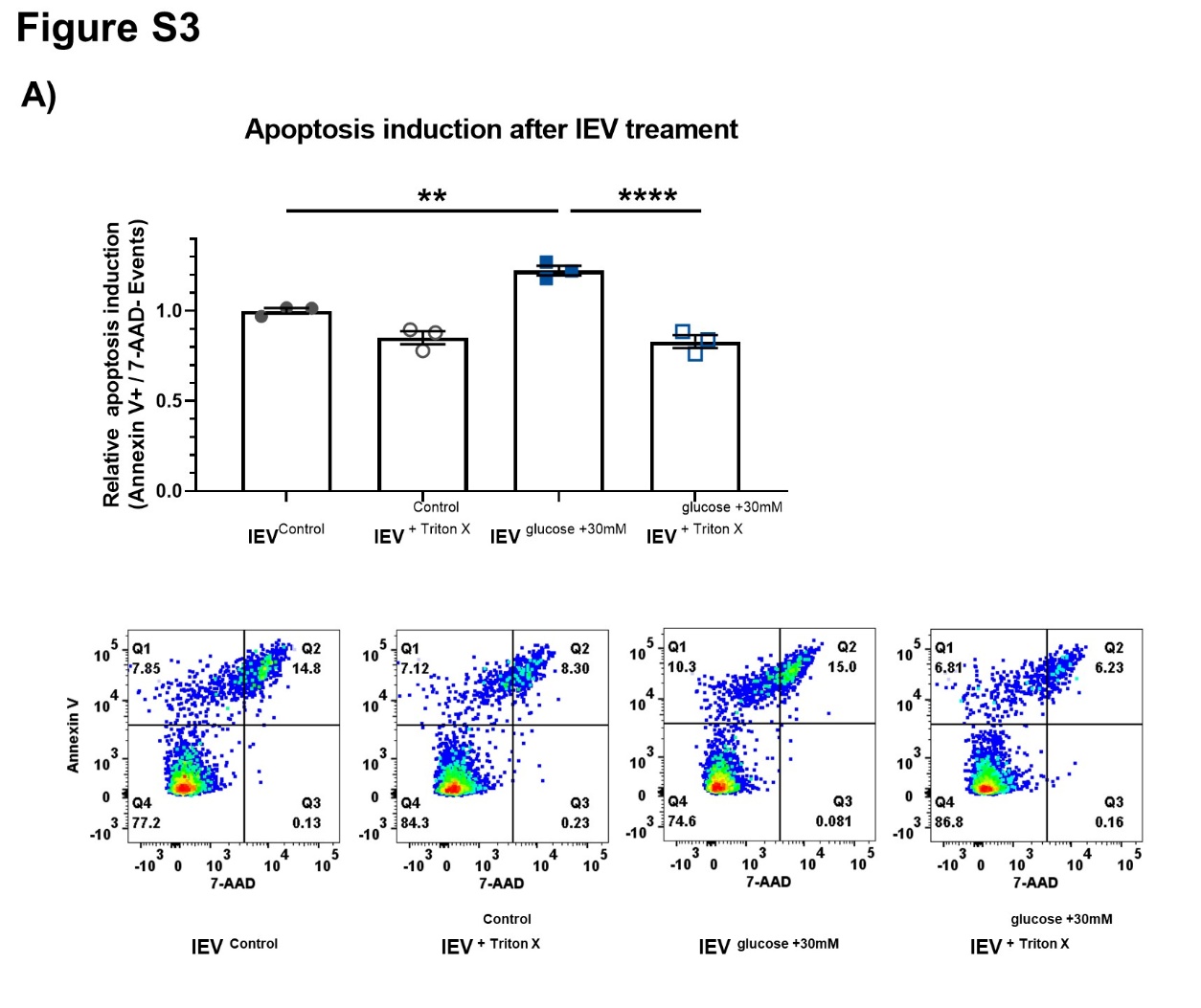
**

### **Figure S3:**

Flow cytometric analysis of the induction of apoptosis in HCAECs that were treated with lEVs from HCAECs after glucose injury, with and without prior degradation of the EVs by use of Triton-X 0.1%; (below) representative dot blots, n=3; **p<0.01, ****p<0.0001. ANOVA + Bonferoni’s multiple comparison test were used.


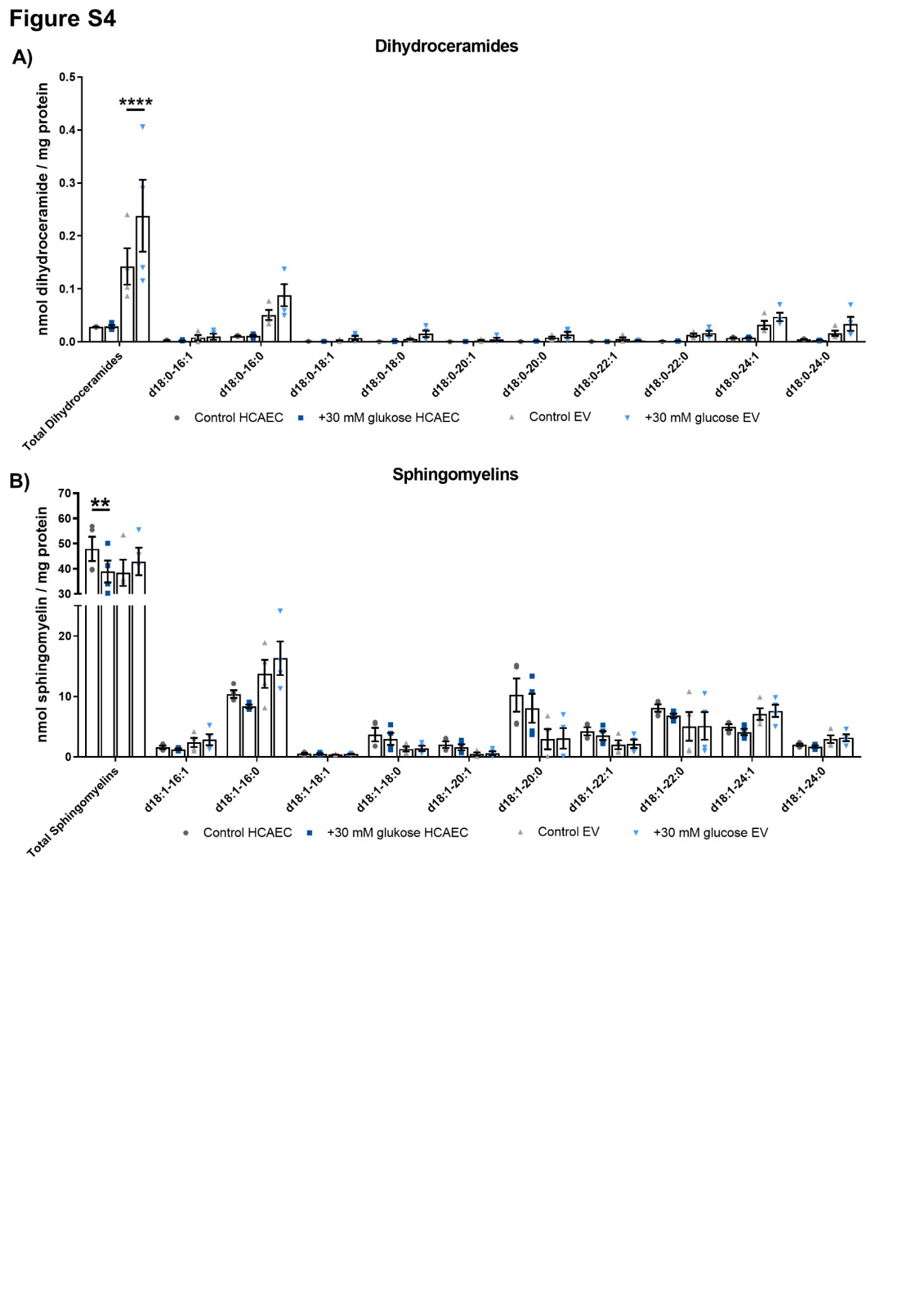


**Figure S4:**

Mass-spectrometric analysis of **A)** dihydroceramides and **B)** sphingomyelins in HCAECs and lEVs after hyperglycemic injury. All data are presented as individual experiments with the mean ± SEM; **p<0.01, ****p<0.0001. 2-way ANOVA + Bonferoni’s multiple comparison test.


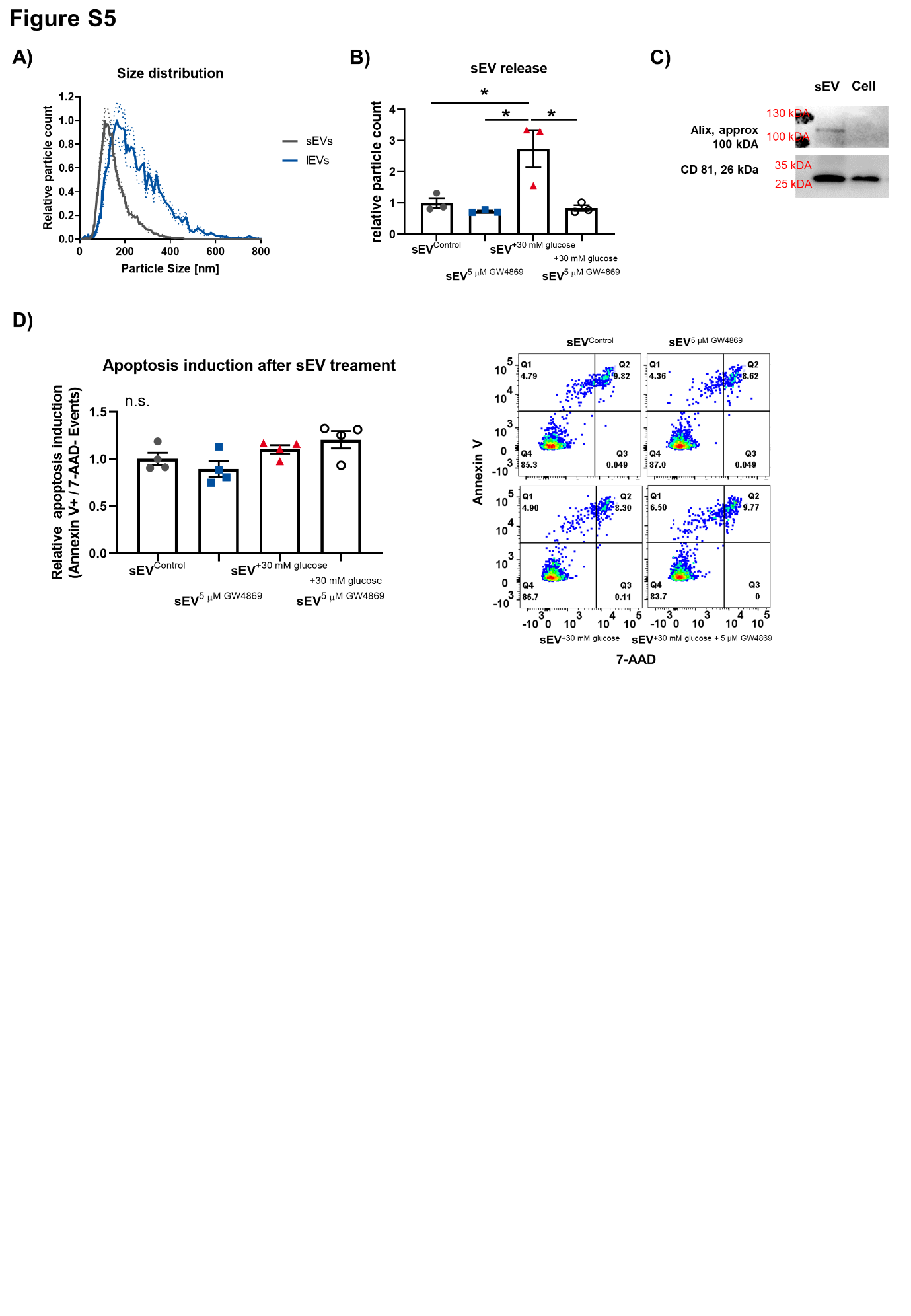


**Figure S5:**

**A+B)** Distribution of sEV and lEV size and sEV release from HCAECs, as analyzed by nanoparticle tracking analysis, n=3. **C)** Characterization of sEVs by immunoblotting for Alix, CD81. **D)** Flow cytometric analysis of the induction of apoptosis in HCAECs after treatment with sEVs from HCAECs that have undergone glucose injury and/or GW4869 treatment with representative dot blots (right side), n=4. All data are presented as individual experiments with the mean ± SEM; n.s. not significant, *p<0.05, ANOVA + Bonferoni’s multiple comparison test were used for B, D.

**
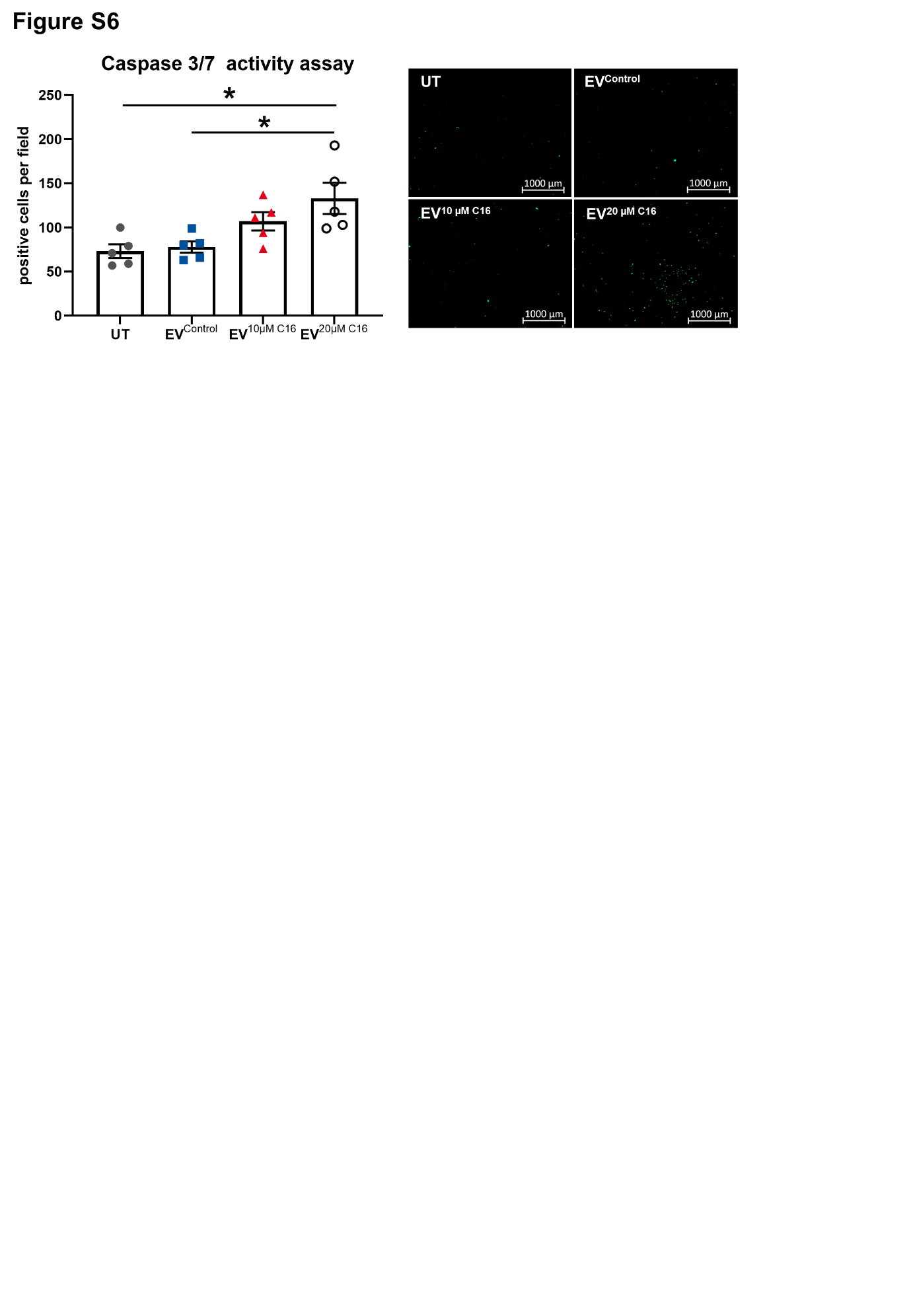
Figure S6:**

Caspase 3/7 activity assay of lEV^C16^-recipient HCAECs with representative images, n=5. All data are presented as individual experiments with the mean ± SEM; *p<0.05, ANOVA + Bonferoni’s multiple comparison test were used.

*
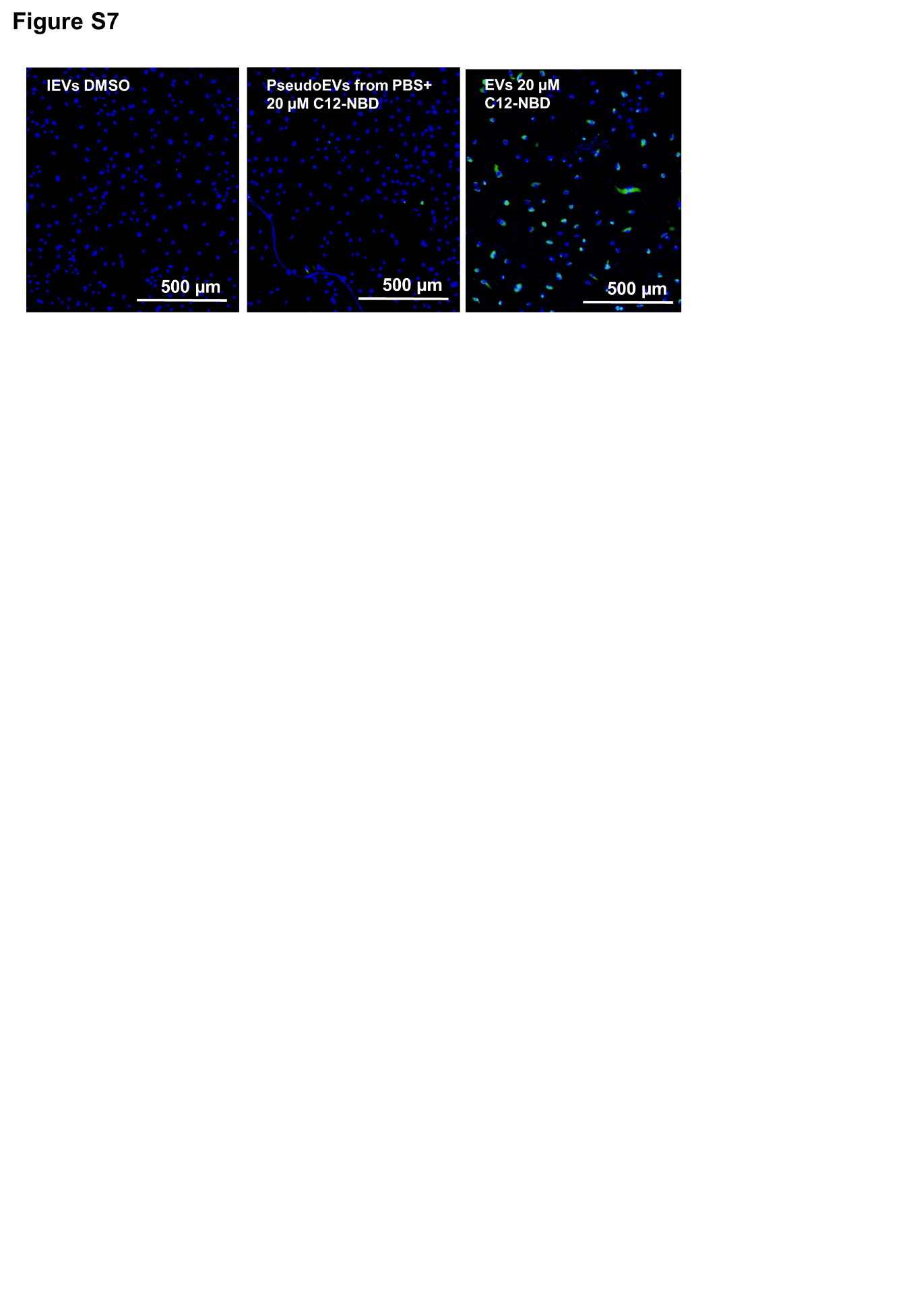
*

**Figure S7:**

NBD-C12 ceramide transfer experiment to exclude the spontaneous formation of lEV-like structures from an aqueous solution of 20 µM NBD-C12 ceramide. Green: NBD-C12 ceramide, blue: DAPI.
